# An adaptive multiarm randomised trial of biomedical and psychosocial interventions to improve convalescence following severe acute malnutrition in sub-Saharan Africa: Co-SAM trial protocol

**DOI:** 10.1136/bmjopen-2024-093758

**Published:** 2025-05-24

**Authors:** Mutsa Bwakura-Dangarembizi, Beatrice Amadi, Benson O Singa, Sofia Muyemayema, Deophine Ngosa, Laura Mwalekwa, Narshion Ngao, Lydia Kazhila, Batsirai Mutasa, Eddington Mpofu, Louisa Mudawarima, Gerard Bryan Gonzales, Shepherd Mudzingwa, Mukumbi Mutenda, Lucia K Keter, Kuda Mutasa, James M Njunge, Helen Jones, Tracy Naomi Phiri, Evans Mudibo, Nivea Chulu, Florence D Majo, Bernard Chasekwa, Aaron Tembo, Churchil Nyabinda, Chris Oduol, Virginia Sauramba, Naume V Tavengwa, Lisa Langhaug, Isabella Cordani, Melanie Smuk, Thomas Jaki, Robert Ntozini, Judd Walson, Kirkby D Tickell, James Berkley, Paul Kelly, Andrew J Prendergast, Co-SAM Trial Team, Gertrude Tawodzera

**Affiliations:** 1 Zvitambo Institute for Maternal and Child Health Research, Harare, Zimbabwe; 2 University of Zimbabwe, Harare, Zimbabwe; 3 Tropical Gastroenterology and Nutrition Group, University of Zambia, Lusaka, Zambia; 4 Kenya Medical Research Institute, Nairobi, Kenya; 5 Tropical Gastroenterology and Nutrition Group, Lusaka, Zambia; 6 KEMRI-Wellcome Trust Research Programme Nairobi, Nairobi, Kenya; 7 KEMRI Wellcome Trust Programme, Kilifi, Kenya; 8 University of Zambia School of Medicine, Lusaka, Zambia; 9 Nutrition, Zvitambo Institute for Maternal and Child Health Research, Harare, Zimbabwe; 10 Data, Zvitambo Institute for Maternal and Child Health Research, Harare, Zimbabwe; 11 Department of Public Health and Primary Care, Ghent University, Gent, Belgium; 12 University of Zimbabwe Clinical Research Centre, Harare, Zimbabwe; 13 Centre for Geographic Medicine Research Coast, KEMRI-Wellcome Trust Research Programme, Kilifi, Kenya; 14 Centre for Genomics and Child Health, Blizard Institute, Queen Mary University of London, London, UK; 15 Tropical Gastroenterology and Nutritional Group (TROPGAN), Lusaka, Zambia; 16 University of Cambridge, Cambridge, UK; 17 Biostatistics, Zvitambo Institute for Maternal and Child Health Research, Harare, Zimbabwe; 18 Department of International Health, Johns Hopkins Bloomberg School of Public Health, Baltimore, MD, USA; 19 Department of Global Health, University of Washington, Seattle, Washington, USA; 20 Childhood Acute Illness and Nutrition Network, Nairobi, Kenya; 21 Clinical Research, KEMRI/Wellcome Trust Research Programme, Kilifi, Kenya; 22 Barts and The London School of Medicine, London, UK

**Keywords:** Mortality, Child, Nutrition

## Abstract

**Introduction:**

Children discharged from hospital following management of complicated severe acute malnutrition (SAM) have a high risk of mortality, readmission and failed nutritional recovery. Current management approaches fail to sufficiently promote convalescence after inpatient nutritional rehabilitation. Novel interventions during the post-discharge period could enhance convalescence to help children survive and thrive.

**Methods and analysis:**

The Co-SAM trial is an adaptive, multicountry, phase III, individually randomised clinical trial, based on the principles that (i) interacting biological and social factors drive multimorbidity in children with SAM, and (ii) both medical and psychosocial interventions may therefore ameliorate underlying causal pathways to reduce morbidity and mortality and improve recovery. Children aged 6–59 months with complicated SAM, who have stabilised and started the transition to ready-to-use therapeutic food (RUTF), will be enrolled and randomised to one of five trial arms (standard-of-care alone; antimicrobials; reformulated RUTF; psychosocial support; or a combination of all strategies). Standard-of-care, which is provided in all trial arms, includes RUTF until nutritional recovery (defined as weight-for-height Z-score >−2, mid-upper arm circumference >12.5 cm and oedema-free since the last study visit), and other management recommended in WHO guidelines. The 12-week antimicrobial package provides daily co-formulated rifampicin and isoniazid (with pyridoxine) and 3 days of azithromycin monthly. The reformulated RUTF, which incorporates medium-chain triglycerides and hydrolysed protein to increase nutrient bioavailability and reduce metabolic stress, is provided at the same dose and duration as standard RUTF. The 12-week psychosocial package includes caregiver problem-solving therapy, educational modules, peer support groups and child play. The combined arm includes all interventions. Children start their intervention package prior to hospital discharge, with follow-up data collection in study clinics at 2, 4, 6, 8, 12 and 24 weeks. The primary composite outcome is death, hospitalisation or failed nutritional recovery within 24 weeks post-randomisation. An interim analysis will allow unpromising arms to be dropped, while the final analysis will be conducted when 1266 children have completed the study. Embedded process evaluation and laboratory substudies will explore the mechanisms of action of the interventions.

**Ethics and dissemination:**

The trial has been approved by ethics committees in Zimbabwe, Zambia, Kenya and UK. Dissemination will be via community advisory boards in each country; Ministries of Health; and dialogue with policymakers including UNICEF.

**Trial registration number:**

Clinicaltrials.gov: NCT05994742; Pan African Clinical Trials Registry: PACTR202311478928378.

**STRENGTHS AND LIMITATIONS OF THIS STUDY**

- A randomised trial of both biomedical and psychosocial interventions will provide high-level evidence for the efficacy of holistic strategies to promote convalescence.
- The psychosocial intervention was co-designed with caregivers of children hospitalised with severe acute malnutrition to ensure it is context-specific.
- The adaptive trial design provides the flexibility to introduce new arms and drop unpromising arms as the trial proceeds.
- The trial is not blinded due to the very different intervention components in each arm.
- Recruitment sites are predominantly urban referral hospitals, so findings may not be generalisable.

## Introduction

 Malnutrition underlies 45% of child deaths and has far-reaching educational, economic and health consequences.^1^ Severe acute malnutrition (SAM), characterised by severe muscle wasting and/or presence of nutritional oedema, affects 13.7 million children globally and is the most life-threatening form of malnutrition.^2^ Community-based management of acute malnutrition using ready-to-use therapeutic food (RUTF) has transformed outcomes for children with uncomplicated SAM, but those presenting with poor appetite or medical complications (categorised as having ‘complicated’ SAM) require hospitalisation and often have poor outcomes despite medical and nutritional management.^3^ Mortality among children with complicated SAM in sub-Saharan Africa remains at 10%–20% in hospital^4^ and 10%–15% in the year post-discharge.^5^

Comorbidities often complicate the management of children hospitalised with SAM. Children with HIV and SAM have threefold higher mortality than children with SAM alone, and this excess risk of death persists despite antiretroviral therapy (ART).^5^ Children with cerebral palsy, hydrocephalus and other neurodisabilities have a high risk of SAM due to difficulties in feeding and additional energy needs. In the recent Health Outcomes, Pathogenesis, and Epidemiology (HOPE)-SAM study, on discharge from hospital, children with cerebral palsy had a sixfold higher risk of mortality than children without cerebral palsy.^5^ Stunting is often coincident with wasting and compounds the risk of mortality and readmission to hospital after initial management of complicated SAM.^6^ A study in Malawi showed decrements in physical and cognitive function were apparent 7 years after hospitalisation with SAM, which may increase long-term non-communicable disease risk.^7^ Taken together, there is substantial evidence that SAM is not a single condition due to lack of food but is rather a complex syndrome, characterised by multimorbidity, and that current long-term outcomes are unacceptably poor.

Following hospital discharge after complicated SAM, children continue to have perturbations of multiple physiological processes, which contribute to poor long-term outcomes.^8 9^ Malnutrition is therefore a marker for deranged metabolic, immune and hormonal pathways, together with acute and chronic infections, enteropathy and inflammation. Interventions focusing on nutritional rehabilitation alone are therefore insufficient, and addressing these complex underlying processes may be critical for effective long-term recovery.^10^ Children are also discharged to a home environment characterised by poverty and multiple caregiver vulnerabilities including depression, low decision-making autonomy, lack of social support, gender-restricted family relations, and competing demands on scarce economic resources which may lead to food insecurity.^11^ Caregivers themselves may have impaired physical and mental health. Addressing biomedical factors in the child alone is insufficient to achieve convalescence without addressing the social determinants of this complex multimorbidity.^12^

**Figure 1:**
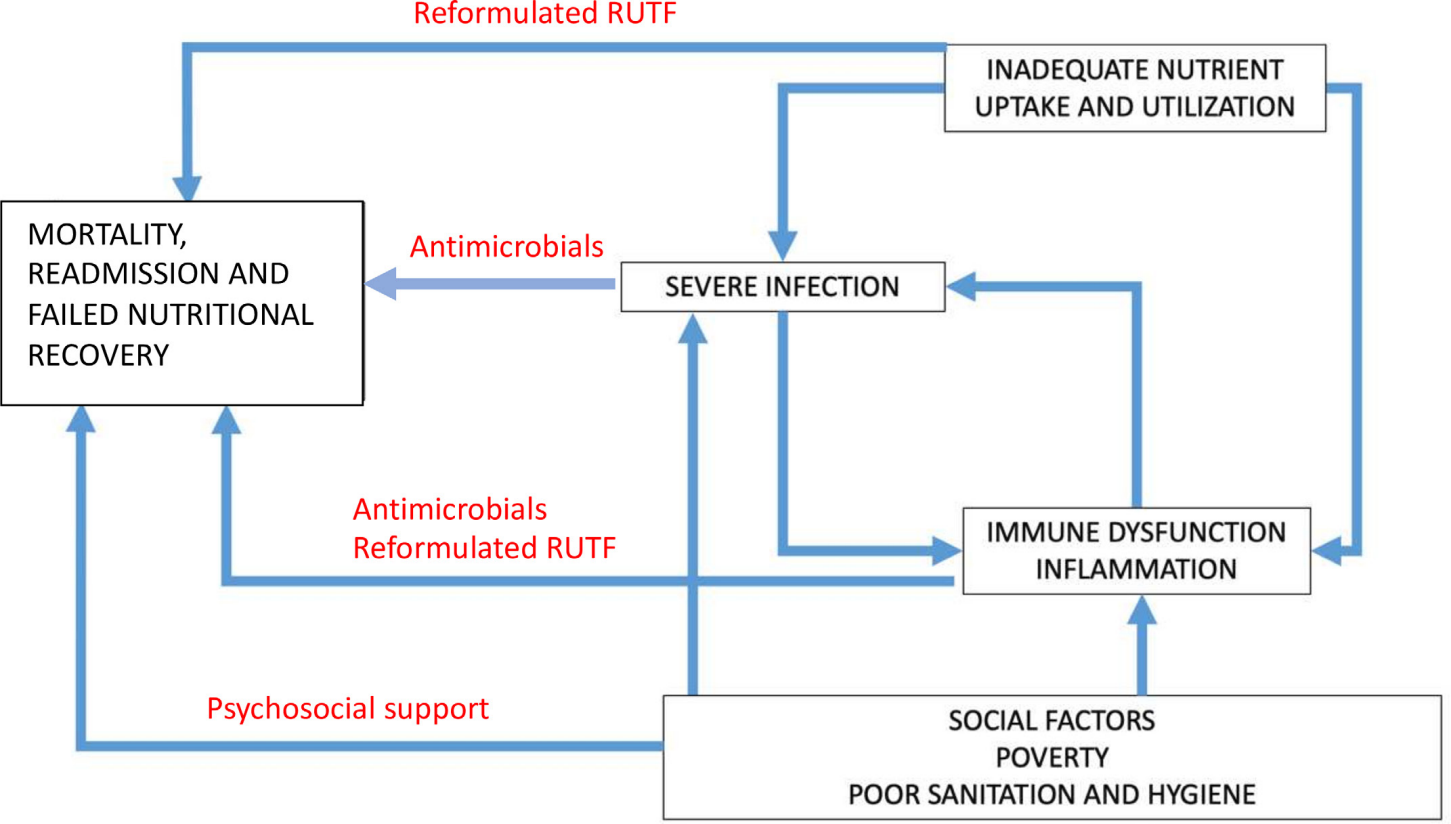
Conceptual framework showing the relationship between poor nutrition, severe infections, social and environmental factors, which drive mortality, rehospitalisation and poor neurodevelopment among children with SAM. The hypothesised action of each intervention is shown in red. SAM, severe acute malnutrition.

We therefore propose a framework of SAM pathogenesis, which identifies potential intervention points to improve outcomes (figure 1). Recent guidelines acknowledge the high-risk period following hospital discharge, when there is an ongoing risk of death, relapse and readmission, but there is a paucity of trials aimed at identifying effective strategies.^3^ Novel interventions during the post-discharge window could enhance convalescence to help children survive and thrive.

### Trial rationale

The adaptive, multicountry, phase III Co-SAM trial is based on the principle that interacting biological and social factors drive multimorbidity in children with SAM, meaning combined biomedical and psychosocial interventions may ameliorate underlying causal pathways to reduce morbidity and mortality and improve recovery. We have developed four intervention packages (antimicrobials; reformulated RUTF; psychosocial support; and a combination of all three) which will each be compared with a control arm, with a primary composite outcome of death, hospitalisation or failed nutritional recovery within 24 weeks post-randomisation. The intervention packages each have a distinct mode of action and rationale:

#### Antimicrobials

Mortality following hospital discharge is predominantly due to untreated, recurrent, iatrogenic or newly acquired infection.^13^ Preventing infections during this high-risk period may prevent relapse, readmission and death. This trial will test a bundle of azithromycin, isoniazid and rifampicin for 12 weeks, targeting key organisms known to be highly prevalent and associated with poor clinical outcomes. Azithromycin is a broad-spectrum antibiotic with additional antimalarial activity and immunomodulatory properties, which may clear subclinical infections, ameliorate enteropathy and reduce systemic inflammation, which all contribute to mortality and hospitalisation.^9^ Community trials in sub-Saharan Africa showed that mass azithromycin administration reduces under-five mortality.^14^ Resistance to azithromycin among pathogens in east and southern Africa remains relatively low, but broader use as a public health intervention increases antimicrobial resistance.^15 16^ Children with SAM are at particularly high risk of tuberculosis (TB).^5^ Ruling out active TB, then providing antituberculous prophylaxis for 12 weeks with isoniazid and rifampicin (plus pyridoxine to prevent neuropathy from isoniazid) to prevent primary or reactivated TB may reduce morbidity and mortality and enhance nutritional recovery.^17^ Rifampicin provides additional gram-positive antibacterial cover, which will effectively treat small intestinal bacterial overgrowth, respiratory and cutaneous infections in combination with azithromycin. However, empirical use of anti-infectives can drive individual and population-level drug resistance and the overall cost/benefit of such approaches must be carefully considered.

#### Reformulated RUTF

Our goal is to reformulate RUTF to increase nutrient bioavailability and reduce metabolic stress and inflammation, thereby, improving nutritional recovery in children with SAM. Providing appropriate nutrients is critical for catch-up growth after SAM.^3^ Therapeutic feeding with RUTF occurs in the setting of profound metabolic dysregulation. During hospitalisation for SAM (especially in children with HIV), there is evidence of increased proteolysis, inflammation and dysregulated lipid metabolism, and a proteomic signature similar to metabolic syndrome.^18 19^ However, about 45%–60% of calories in RUTF are supplied in the form of long-chain triglycerides, which may contribute to the metabolic stress, and hence, to the poor outcomes among children with complicated SAM. The reformulated RUTF will supply some of the lipids in the form of medium-chain triglycerides (MCT), which may reduce metabolic stress, as MCTs are readily absorbed and metabolised by the liver. Evidence of higher gastric pH in children with SAM may reduce the ability to digest proteins^20^; hence, the reformulated product will provide amino acids in the form of pre-hydrolysed milk proteins to increase bioavailability. The product underwent research and development by the manufacturer (Nutriset, France), in collaboration with the trial investigators, to produce the final composition shown in online supplemental table 1. We conducted a pilot affective sensory test to explore the acceptability of the reformulated RUTF. A sachet of the reformulated RUTF was given to 25 children with complicated SAM ready to be discharged from hospital in Lusaka, Zambia. All children were eager to consume the reformulated RUTF, most finishing the sachet in less than 1 hour. Since we did not observe any sign of product rejection, the study team concluded that the RUTF was acceptable. A formal acceptability trial was not performed. The reformulated RUTF is expected to go into production in May 2025 and be introduced into the trial in July 2025.

#### Psychosocial intervention

Feasible, sustainable interventions to improve the psychosocial environment could directly enhance caregiver capabilities, promote child play and increase uptake of medical care, which together would help convalescence. Malnutrition impairs child neurodevelopment, and previous trials have shown benefits from child stimulation^21 22^; however, none has integrated play with strategies to enhance caregiver well-being, which may improve sustained delivery of the intervention at home. Poor physical and mental health, lack of time, reduced agency, and scarce resources make the implementation of such strategies challenging.^11^ Depression is highly prevalent in caregivers of children with SAM and further impairs child growth and recovery.^23 24^ Caregivers are also affected by multimorbidity and immersion in the same adverse environment. Providing caregivers with tailor-made peer support, problem-solving skills and a brief psychological intervention to reduce depression, through trained lay workers with lived experience of SAM, may facilitate uptake of child play to enhance recovery.

The psychosocial intervention being tested in this trial comprises three components, which were co-designed with caregivers in Zimbabwe^25^:

1. *The Friendship Bench* was developed in Zimbabwe as a low-cost psychological intervention using problem-solving therapy (delivered by trained lay workers) and peer-to-peer support to address common mental disorders.^26^ There is a strong evidence base for its use in urban settings.^26^ Peer support groups meet every 1–2 weeks and focus on communal problem-solving and establishing income-generating activities.
2. *Care for Child Development* is a UNICEF package that helps families build stronger relationships and solve problems in caring for their child at home, through play and communication activities to stimulate children, using a series of age-appropriate interactive modules delivered by a lay worker using ‘flash’ cards.^27^ It has been used in other African contexts and has good acceptability.
3. Educational and behaviour-change messages around better nutrition; play for children with SAM; stigma, HIV financial planning; causes of SAM; and health-seeking behaviours. These were developed with caregivers of children with SAM through a series of co-design workshops, ensuring they are contextually relevant.

#### Combined intervention

Combining antimicrobials, reformulated RUTF and child play/caregiver support may have a greater efficacy than any single intervention alone, due to the multifactorial drivers underlying mortality in SAM.^28^ Since each intervention has a distinct mechanism of action, a holistic biomedical and psychosocial support ‘package’ may address several pathways underpinning poor long-term outcomes. There may also be synergies between the components: for example, improving enteropathy and reducing inflammation through antimicrobials may increase absorption of nutrients in the reformulated RUTF, while tackling caregiver mental health may increase uptake of the biomedical interventions.

### Objectives and hypothesis

The primary trial objective is to evaluate the efficacy of each intervention against standard-of-care (control arm) in children being discharged from hospital following complicated SAM in Zimbabwe, Zambia and Kenya. The hypothesis is that each intervention, provided for 12 weeks to children being discharged from hospital, will reduce 24-week mortality, hospitalisation or failed nutritional recovery in children with SAM compared with standard-of-care. Secondary trial objectives are (i) to document how interventions are implemented and perceived in practice through process evaluation; and (ii) to define the mechanism of action of the individual and combined intervention packages, through process evaluation to document fidelity of implementation and uptake of the psychosocial intervention, and laboratory substudies to explore the impact of the biomedical interventions on metabolic and inflammatory pathways.

## Methods and analysis

### Trial setting

The Co-SAM trial is being undertaken at nine sites in three countries. In Zimbabwe, where the prevalence of wasting is 2%,^29^ three hospitals in Harare (Sally Mugabe Children’s Hospital, Parirenyatwa Hospital and Chitungwiza Central Hospital) serve a predominantly urban and peri-urban population, although some children are referred for inpatient care from rural districts. In Zambia, the University Teaching Hospital Children’s Hospital (UTHCH) in Lusaka provides secondary level services to the city of Lusaka (population 1.9 million, census 2022) and tertiary level services to the whole country (population 19 million). The child malnutrition ward in UTHCH provides inpatient care for children with complicated SAM, most of whom are drawn from high-density urban and peri-urban residential areas of the city. Matero Level 1 hospital provides primary and secondary level care for the high-density areas of north-west Lusaka. The prevalence of SAM in Zambia was 1.5% in 2018.^30^ There are four hospitals in Kenya: Migori County Hospital and Homa Bay County Teaching and Referral Hospital serve a rural area in Western Kenya; Coast General Teaching and Referral Hospital, Mombasa serves an urban and peri-urban population on the coast; and Mbagathi sub-County Hospital predominantly serves a population living in informal settlements in Nairobi. The prevalence of acute malnutrition among under-5 children in Kenya in 2022 was 4.9%.^31^

### Trial design

**Figure 2:**
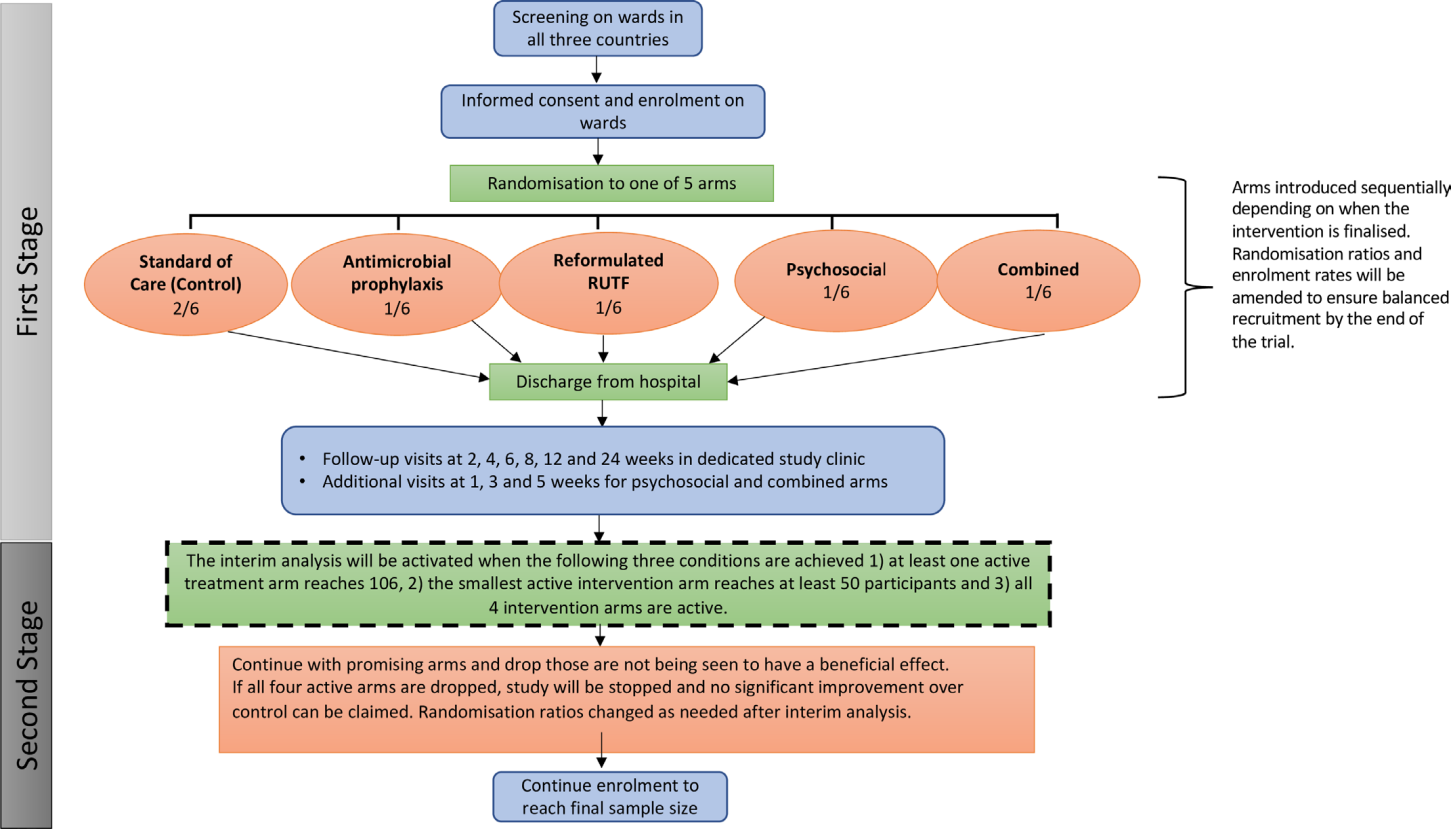
Trial schema showing the adaptive design, with arms introduced as they become available. The trial will start with enrolment into three arms (standard-of-care, antimicrobials and psychosocial), with introduction of the reformulated RUTF and combined arms once the reformulated product is developed and approved. An interim analysis will be undertaken once the three conditions listed are met, with unpromising arms dropped and randomisation ratios altered as needed to complete the trial with the remaining arms. RUTF, ready-to-use therapeutic food.

The trial protocol and case report forms are available at https://osf.io/824av/. Co-SAM is a five-arm, individually randomised clinical trial among children hospitalised with complicated SAM (figure 2). Children who have stabilised and started the transition to RUTF will be enrolled and randomised to one of the trial arms in hospital and will start the 12-week intervention prior to discharge. The trial will test the superiority of each intervention arm over a standard-of-care arm, using a 2:1:1:1:1 randomisation ratio in favour of standard-of-care. There will be no blinding or placebo as each trial arm has very different components. The trial is adaptive, meaning: (i) one country can start before another; (ii) trial arms will be introduced once each intervention is ready for testing; and (iii) an interim analysis will enable interventions which are unpromising to be dropped, based on pre-specified criteria.

### Trial procedures

The trial schedule is shown in online supplemental table 2.

#### Screening and enrolment

Children with complicated SAM will be screened and enrolled from hospitals once they have stabilised after their admission. Children of either sex will be eligible if they are age 6–59 months, hospitalised with complicated SAM (per WHO definitions),^3^ have started the transition from therapeutic milk feeds to RUTF, and have a caregiver who is willing and able to give informed consent and to attend all study visits. Children will not be eligible if they have any acute or chronic condition which means that receipt of study interventions, or participation in the trial, would not be advisable.

#### Baseline procedures

Following enrolment, baseline procedures will be conducted while the child is still hospitalised, prior to randomisation. Demographic, child and caregiver data will be collected, and children will undergo clinical examination, review of medical history and current medications, HIV testing (if not done previously), TB diagnostics (if not done previously) and anthropometry. Blood will be collected to measure baseline liver function and to store plasma and leucocytes. Stool will be collected and stored for subsequent measurement of enteropathy biomarkers.

#### Randomisation

The trial will begin with three arms active (control, antimicrobial and psychosocial) with a randomisation ratio of 2:1:1. The other two arms will be introduced when the reformulated RUTF has been developed (anticipated July 2025), and the ratio will be amended to rebalance towards the designed 2:1:1:1:1 ratio (standard-of-care, antimicrobial, reformulated RUTF, psychosocial and combined arms, respectively). The randomisation ratio may be altered a third time after the interim analysis if an arm is dropped to ensure a 2:1 ratio of standard-of-care vs each remaining intervention arm. Participants will be prospectively randomised sequentially using random permuted blocks with random block size, stratified by hospital site. The trial database will contain a secure, computer-generated, sequentially numbered randomisation list prepared by the trial statistician. If the caregiver has another child who has already been randomised in the trial, subsequent children will be automatically allocated to the same intervention. If a child has already been enrolled, s/he will not be eligible to re-enrol during a subsequent hospital admission.

### Interventions

Interventions will be started as soon as possible after randomisation while the child is still in hospital and continued for 12 weeks post-randomisation through outpatient visits. All children will receive standard-of-care interventions, together with arm-specific interventions.

#### Standard-of-care arm (control)

Children will receive best available current care, based on WHO guidelines.^3^ All children will receive RUTF (Plumpy’nut; Nutriset, France), dosed according to weight bands and continued until nutritional recovery (weight-for-height Z-score >−2, mid-upper arm circumference >12.5 cm, and oedema-free since the last study visit). All children living with HIV will start ART and cotrimoxazole according to national guidelines. All medications required for standard care (eg, antibiotics, TB treatment) will be provided. Children will be seen in a dedicated study clinic, with medical care provided as needed, and will not additionally need to attend their local outpatient therapeutic programme.

#### Antimicrobial arm

Children will receive all standard-of-care interventions, plus a 12-week course of isoniazid and rifampicin (with pyridoxine) as TB prophylaxis; and a 3-day course of azithromycin monthly, as antibacterial/antimalarial prophylaxis. Children will receive their first doses of medications on the ward. Rifampicin and isoniazid will be provided as dispersible, co-formulated tablets to be given daily according to WHO weight bands; pyridoxine will be provided as a standard 12.5 mg dose daily; and azithromycin will be given as a 10 mg/kg dose for 3 days each month (ie, 9 doses over 12 weeks) as liquid suspension using an oral syringe. Any children with contraindications to components of the bundle will omit that drug and receive all other medications. Any children receiving therapy for TB disease will complete their treatment course prior to starting the 12-week prophylaxis strategy.

#### Reformulated RUTF

Children will receive all standard-of-care interventions but will replace standard RUTF with reformulated RUTF. The number of daily sachets will use the same weight bands as for standard RUTF and will be continued until the same endpoint of nutritional recovery (defined above). The study team will help the caregiver introduce the reformulated RUTF prior to discharge and will ensure that no standard RUTF is supplied from the ward.

#### Psychosocial

The caregiver will meet an intervention facilitator (IF)—a lay worker who previously had a child hospitalised with SAM, who is trained and supervised by the research team to deliver the psychosocial package. The IF will deliver an introductory session in hospital, then see the caregiver-child pair weekly in the study clinic to deliver six face-to-face sessions. Each session comprises three components: (i) problem-solving therapy using a structured approach to identify problems and generate solutions; (ii) an interactive education module; and (iii) an age-appropriate play session. The same IF will deliver the subsequent sessions at each weekly outpatient visit from weeks 1–6.

#### Combined arm

The caregiver-child pair will start all three trial interventions, as outlined above.

The study team will provide the caregiver with an arm-specific booklet, providing an overview of their daily intervention; tools for recording adherence; 24-hour contact details for the study team; and their study clinic appointment dates. Prior to discharge, a study team member will collect household contact numbers, location and contact details of two neighbours, friends or relatives, and any plans for relocation.

#### Follow-up procedures

Children will attend a dedicated study clinic at weeks 2, 4, 6, 8 and 12 post-randomisation (with additional visits at 1, 3 and 5 weeks for the psychosocial and combined arms), with reimbursement provided for transport and time spent in the study clinic. Children are seen for additional clinical visits as determined by the trial team. At study visits, data will be collected on illness and hospitalisation (at study hospitals and other hospitals), and anthropometry will be conducted. Blood and stool will be collected at weeks 2 and 12, with repeat liver function testing at week 2 to identify possible hepatotoxicity from study drugs. RUTF will be supplied in all arms for at least 2 weeks, and then continued until the child achieves nutritional recovery (defined as a weight-for-height Z-score >–2, mid-upper arm circumference >12.5 cm, and oedema-free since the last study visit), using either standard RUTF or reformulated RUTF, depending on trial arm. If the child has not achieved nutritional recovery by week 12, or relapses before week 24 following nutritional recovery, s/he will be defined as reaching the primary outcome (failed nutritional recovery; see table 1); RUTF will be continued to a maximum of 16 weeks, and the child will be investigated for underlying causes. If the child achieves nutritional recovery but subsequently relapses, s/he will be defined as reaching the primary outcome, and RUTF will be restarted. In the antimicrobial arm, the study team will elicit any side effects, assess adherence by asking the caregiver to recall missed doses, and dispense medication at each visit to week 12. In all arms, the study team will check if children with HIV have started ART and cotrimoxazole; if not, ART initiation will be facilitated through referral to national programmes. In the psychosocial arm, the IF will undertake problem-solving therapy with the caregiver, deliver a module of behaviour-change material and undertake play activities with the child. After at least three individual sessions, the caregiver will be referred to a peer support group, which will meet weekly for six sessions. This group is led by an IF, with a focus on group problem-solving and income-generating activities. In the combined arm, all three interventions will be delivered at each study visit.

**Table 1:** Trial outcomes

Primary outcome	
Death or first hospitalisation or failed nutritional recovery within 24 weeks post-randomisation	All-cause mortality or overnight admission to a health facility for any reason*, or failed nutritional recovery† within 24 weeks of randomisation

Secondary outcomes	
Change in weight-for-height Z-score between baseline and 24 weeks post-randomisation	Change in weight-for-height Z-score between baseline and 24 weeks post-randomisation according to age-appropriate and sex-appropriate WHO reference standards
Change in mid-upper arm circumference between baseline and 24 weeks post-randomisation	Change in size of mid-upper arm in centimetres between baseline and 24 weeks
Change in weight-for-age Z-score between baseline and 24 weeks post-randomisation	Change in weight-for-age Z-score between baseline and 24 weeks post-randomisation according to age-appropriate and sex-appropriate WHO reference standards
Change in height-for-age Z-score between baseline and 24 weeks post-randomisation	Change in height-for-age Z-score between baseline and 24 weeks post-randomisation according to age-appropriate and sex-appropriate WHO reference standards
Suspected or confirmed tuberculosis, pneumonia, diarrhoea or malaria through 24 weeks post-randomisation	Physician-diagnosed suspected or confirmed infection, as defined by WHO guidelines, between baseline and 24 weeks post-randomisation

Tertiary outcomes	
Change in anthropometry between baseline and 4 weeks post-randomisation	Change in WHZ, WAZ, HAZ and MUAC between baseline and 4 weeks post-randomisation
Change in anthropometry between baseline and 12 weeks post-randomisation	Change in WHZ, WAZ, HAZ and MUAC between baseline and 12 weeks post-randomisation
Change in caregiver mental health between baseline and 24 weeks post-randomisation	Change in Shona Symptom Questionnaire score (and proportion meeting cut-off score >8) between baseline and 24 weeks post-randomisation
Mechanistic laboratory endpoints	Exploratory endpoints based on comparing lipidomic, metabolomic, inflammatory and proteomic signatures between arms

* Includes cases where there was a clinical plan to hospitalise the child, which was refused by the caregiver.

† Defined as either: (i) persistent WHZ <–2 or MUAC <12.5 cm or bilateral pedal oedema at week 12; or (ii) WHZ <–2 or MUAC <12.5 cm or bilateral pedal oedema at any time between baseline and week 24 post-randomisation in a child who had previously recovered.

HAZ, height-for-age Z-score; MUAC, mid-upper arm circumference; WAZ, weight-for-age Z-score; WHZ, weight-for-height Z-score.

At 24 weeks post-randomisation, children will be seen for an endline research visit to collect primary and secondary outcome data, including repeat anthropometry. Children who fail to attend the 24-week visit will be seen at home, or caregivers will be contacted by phone, to collect intention-to-treat data on the primary outcome. No trial interventions will be provided beyond week 24. If children require ongoing care, they will be linked to local Ministry of Health services.

### Trial outcomes

Trial outcomes are listed in table 1.

### Laboratory analyses

Stool will be collected into sterile containers, then aliquoted for storage at −80°C. Whole blood will be centrifuged to separate plasma which will be aliquoted and stored at −80°C. The remaining cell pellet will be resuspended in red cell lysis buffer to deplete red cells and fix leucocytes, followed by a wash step, resuspension in freezing medium and aliquot storage at −80°C. Planned laboratory analyses on archived samples include targeted and untargeted lipidomics, metabolomics, proteomics and biomarker assays. These analyses aim to elucidate key biological pathways linked to poor post-discharge convalescence including metabolic, lipid and hormonal perturbations, inflammation and enteropathy, and the impact of the trial interventions on these mechanisms. Future substudies will capitalise on the biobank generated during the trial and perform exploratory analyses including the characterisation of cellular immune profiles before and after trial interventions.

### Data management

Study participants will be managed using an in-house, custom-developed secure database with pharmacy and laboratory management functions. Case report forms (CRFs) will be a combination of paper and electronic (eCRFs) forms captured using Research Data Capture (RedCap). All CRFs and eCRFs will have skip patterns, plausibility checks and will flag missing variables. All CRFs will be completed by clinical research staff and checked by data managers at each site. All identifiable data will be stored on paper in locked cabinets at each site accessible only to the study team and separate to CRFs containing de-identified data, which are labelled with unique study identifiers. Completed eCRFs will be uploaded to the trial server. Internal quality assurance systems will monitor data completion, data transfer and storage of CRFs regularly. A central team of data managers will provide automated queries and reviews of incoming data daily through a dashboard system across sites. Any missing or implausible data will be highlighted for site teams to resolve within stipulated timeframes.

### Sample size

For the primary analysis, a total sample size of 1266 participants across all three countries, with a final ratio of 2:1:1:1:1 in favour of standard-of-care, provides 80% power to detect a 30% reduction in the composite primary outcome of death or hospitalisation or failed nutritional recovery within 24 weeks, assuming a control arm event rate of 35% with overall one-sided alpha of 0.10 (ie, family-wise error),^32^ allowing for 5% loss to follow-up if all arms start at the same time. The control arm event rate was based on published data on mortality, readmission and nutritional recovery from the HOPE-SAM study^5 33^ in Zimbabwe and Zambia, and the Childhood Acute Illness and Nutrition network,^13 34^ which included sites in east Africa. Children who are enrolled but not randomised for any reason will be replaced.

### Statistical analysis

A pre-specified statistical analysis plan will be posted online at https://osf.io/824av/ prior to any analyses.

#### Interim analysis

One interim analysis will be undertaken once the following three conditions are achieved: (1) at least one active intervention arm reaches 106 participants; (2) the smallest active intervention arm reaches at least 50 participants; and (3) all four intervention arms are active. A logistic regression model accounting for country, age, sex and baseline oedema with indicators for each active treatment will be fitted on the composite primary outcome. If the test statistic falls below the lower (futility) bound of zero in an arm, indicating that there is no benefit of that arm over control, then that arm may be discontinued. Similarly, if the test statistic of an arm exceeds the upper (efficacy) bound (2.693), then that arm may be discontinued as it clearly suggests substantially lower mortality, hospitalisation or failed nutritional recovery than the control group. Active arms that have test statistics between the lower and upper bound will be continued, as they suggest potential benefit. The boundary estimates (0 and 2.693) have been calculated to control for the family-wise error. All decisions about dropping arms will be made in conjunction with the Data Monitoring and Ethics Committee, which comprises four independent members, and Trial Steering Committee, which comprises five independent members.

#### Final analysis

The primary analysis will include all participants as randomised (‘intention-to-treat’), irrespective of whether they received their allocated treatment. Both treatment effects and 95% CIs will be reported for all primary, secondary and subgroup analyses. The per-protocol analysis is a secondary analysis and will include all children without major protocol violation who had the primary outcome measured. Final analyses will be undertaken once 24-week data are available for at least 211 children in each active arm selected to continue to the second stage, and at least 422 control children. A logistic regression model with country, age, sex and oedema status as covariates, and a treatment indicator for each intervention, will be fitted. From the model, test statistics will be computed to compare each active arm to the control arm. If the test statistic exceeds the critical value of 1.904, then the corresponding arm will be identified as a promising intervention. Secondary outcomes will be evaluated using the same principles and critical values as the primary analysis. A linear regression model will be used for continuous outcomes (transformed if required); a logistic regression model will be used for binary outcomes; and a proportional hazards model will be used for time-to-event outcomes. If there are substantial missing data, the mechanism will be explored and appropriate methods (eg, multiple imputation) implemented to remove potential inference bias. Statistical code for the main analyses will be made available with the primary publication. The final trial dataset will be made open access at ClinEpiDB after the analyses in the protocol have been reported.

#### Process evaluation

The trial will explore how interventions are implemented and perceived in practice to support interpretation of outcome data. The process evaluation will include periodic structured and informal observation of intervention activities and interactions, and organised reflection and group debrief sessions with implementers and caregivers, paying particular attention to actual and potential unintended negative outcomes and opportunity costs for household members, implementers and broader health services and health systems.

### Safety

#### Risks and benefits

We believe novel interventions are justified in SAM as it is a high-risk condition, and current practice is insufficient to reduce mortality to acceptable levels. There is clinical equipoise for all the proposed interventions.

Although there are potential side effects from antimicrobials, these are generally well-tolerated drugs with a long track record of use and excellent safety profile. There are several reasons why this trial will test universal prophylaxis, rather than targeted prophylaxis in a subgroup of children at high risk of infections. First, the population risk for mortality and readmission in children with SAM is extremely high. Second, we are currently unable to identify the children at highest risk of mortality following hospitalisation, meaning it is not possible to identify a subgroup most likely to benefit. Third, children are already treated in a targeted manner for symptomatic infections which can be diagnosed in the clinic, yet mortality remains unacceptably high, meaning new preventive approaches are required. Fourth, the trial is testing a pragmatic approach which could easily be scaled up. Finally, this approach has been safely tested in other trials with a very similar antimicrobial bundle.^17^ If we find evidence of efficacy, the embedded mechanistic studies using biological samples may enable us to identify a subgroup of children to target in the future. At a population level, more widespread use of antibiotics must be balanced against the risk of promoting antimicrobial resistance (AMR). The recent MORDOR trial of azithromycin, which was associated with modest increases in AMR to macrolides,^35^ led to a WHO recommendation that mass administration of azithromycin was justifiable in settings of high child mortality.^36^ Given the high-risk population of children with SAM (10% mortality over 12 months^5^), we believe this is a justifiable risk.

Ready-to-use therapeutic food is well tolerated and universal in guidelines globally for SAM.^3^ Although it contains peanuts and milk powder, there have been no reported cases of anaphylaxis; however, we will only enrol children who are already tolerating RUTF. The reformulated RUTF alters the composition of several nutrients but is not expected to affect the safety profile of the product. Our reformulation is grounded in observations of the distinct metabolic milieu in children with SAM,^18^ and it is likely that current recommended formulations are higher risk than the proposed reformulated product, which is designed to be more digestible and anti-inflammatory.

The psychosocial intervention is a low-risk intervention designed to provide play therapy, caregiver support and income-generating opportunities with peers. Screening for depression and anxiety may reveal caregivers with common mental disorders, but we will provide the best available, evidence-based intervention through the Friendship Bench. We will evaluate whether the time burden of this intervention for caregivers leads to unintended negative effects.

#### Safety monitoring

All adverse events will be reported by research staff and reviewed by clinicians at each site for seriousness, relatedness, severity and expectedness. All serious adverse events will be reported to local regulators, the Data Monitoring and Ethics Committee and the trial sponsor.

### Patient and public involvement

This trial is directly informed by prior clinical and social science research in the same communities.^11 37^ All three countries have been engaged in hospital and community-based studies of SAM for two decades and have demonstrated the critical role of the household and broader community in health-seeking behaviour and recovery following hospital discharge. The interventions were informed by in-depth interviews with caregivers of children with complicated SAM, which identified existing barriers to convalescence once children are discharged from hospital.^11 37^ Caregivers in Zimbabwe identified lack of social support, economic hardship and limited time for childcare as key issues they faced; they felt strongly that cash transfers were not a feasible intervention approach. Qualitative research in Kenya identified multiple caregiver vulnerabilities including depression, low decision-making autonomy, lack of social support, gender-restricted family relations, and competing demands on scarce resources. The psychosocial package was iteratively co-designed with caregivers of children with SAM in Zimbabwe and has been piloted in 30 families, demonstrating feasibility and acceptability.^25^ Ongoing social science work in each country will inform the context in which the interventions will be delivered. In each country, a community advisory board has been established to provide a forum for two-way dialogue about the study.

## Ethics and dissemination

### Informed consent

Written informed consent will be provided by the caregivers of participants in local languages (English version in online supplemental file 1). Caregivers with poor literacy who have understood a verbal explanation of the study can provide a thumbprint on the consent form in the presence of an independent witness.

### Dissemination of results

In each country, results will be disseminated via community advisory boards and Ministries of Health, as well as policymaker events to discuss the implications of the trial findings. Trial results will be disseminated at international conferences, and open-access peer-reviewed journals, as well as via the Co-SAM website.

### Time frame and study status

The trial has undergone ethical approval in Zimbabwe (Joint Research Ethics Committee for the University of Zimbabwe, Faculty of Medicine and Health Sciences & Parirenyatwa Group of Hospitals (206/2023); Medical Research Council of Zimbabwe (MRCZ/A/3064); and Medicines Control Authority of Zimbabwe (CT262/2023)); Zambia (University of Zambia Biomedical Research Ethics Committee (4266–2023); Zambia Medicines Regulatory Authority (CT135/24); and the National Health Research Authority (NHREB002/25/03/2024)); Kenya (Kenya Medical Research Institute (KEMRI) Scientific and Ethics Review Unit (KEMRI/CCR/CSC/0316/4808); Pharmacy and Poisons Board (ECCT/24/02/07) and the National Commission for Science, Technology & Innovation (NACOSTI/P/25/414674)); and the UK (Oxford Tropical Research Ethics Committee (44-23)). The trial Sponsor is Queen Mary University of London (EDGE ID 150522). Any protocol amendments will only be implemented after approval by regulators in each country and the Sponsor. The trial started enrolment in Zimbabwe and Zambia in July 2024 and in Kenya in March 2025. By 15 May 2025, a total of 291 children had been enrolled to the trial. The estimated date of the last participant enrolment is September 2027.

## Discussion

Complicated SAM is a high-risk condition with an ongoing risk of mortality, relapse and readmission in the year following hospitalisation.^5^ New WHO guidelines on SAM include a good practice statement about the need to provide additional support for children during this high-risk window of vulnerability, but there is a very limited evidence base.^3^ Pathogenic perturbations continue for at least a year after management of complicated SAM,^9^ which informed our choice of biomedical interventions designed to ameliorate the metabolic, enteropathic and inflammatory processes that underlie SAM. In addition, the socioeconomic, caregiver and home factors that contribute to poor recovery from SAM likely need to be addressed through a psychosocial intervention. The Co-SAM trial therefore aims to generate evidence for biomedical and psychosocial interventions, delivered separately or together, to identify new strategies to improve outcomes in this high-risk condition, so that all children can survive and thrive.

## Supplementary material

10.1136/bmjopen-2024-093758 online supplemental file 1

10.1136/bmjopen-2024-093758 online supplemental file 2

10.1136/bmjopen-2024-093758 online supplemental file 3
