# Supplementary material for: An adaptive multiarm randomised trial of biomedical and psychosocial interventions to improve convalescence following severe acute malnutrition in sub-Saharan Africa: Co-SAM trial protocol
Source: BMJ Open. 2025 May 24;15(5):e093758. doi: 10.1136/bmjopen-2024-093758 (PMC12104919; doi:10.1136/bmjopen-2024-093758)
Supplement: online supplemental file 1 [file bmjopen-15-5-s001.pdf]

No. 16 Lauchlan Avenue
Meyrick Park, Mabelreign
Harare, Zimbabwe
Tel:+263 (242) 306028/306654
E-mail: administrator@zvitambo.com

INFORMED CONSENT FORM - CAREGIVERS **Participant SID: ZW** ☐☐☐☐

An adaptive multi-arm trial to improve clinical outcomes among children recovering from complicated severe acute malnutrition (Co-SAM)

A study of different treatments to improve recovery after severe acute malnutrition

Principal Investigator: Prof Mutsa Bwakura Dangarembizi

Phone number(s) +263 (242) 306028 or 306654

What you should know about this research study:

- We give you this consent form so that you may read about the purpose, risks, and benefits of this research study.
- The main goal of research studies is to gain knowledge that may help future patients.
- We cannot promise that this research will benefit your child. Just like regular care, this research can have side effects that can be serious or minor.
- You have the right to refuse to allow your child to take part, or you can agree for your child to take part now and change your mind later.
- Whatever you decide, it will not affect your child's regular care.
- Please review this consent form carefully. Ask any questions before you make a decision.
- Your choice to allow your child to participate is voluntary.

PURPOSE

You are being asked if you and your child would like to participate in a research study. The aim of the study is to investigate how we can improve recovery and prevent readmission to hospital for children with Severe Acute Malnutrition (SAM). We know that children with SAM are at an increased risk of becoming unwell again, relapsing with SAM, or even passing away after leaving hospital. In this study, we will compare the care that children usually receive (standard care) with additional new treatments, given on their own or combined. We want to know which intervention(s) will be most practical and have the greatest benefit in helping children recover from SAM and stay well at home. The study is being overseen by researchers from Zvitambo as well as Queen Mary University of London, UK.

WHY HAS MY CHILD BEEN CHOSEN?

Your child has Severe Acute Malnutrition and has been admitted into hospital. We would like to include your child in a trial of some new treatments.

HOW MANY CHILDREN WILL BE IN THE STUDY?

The study is being conducted in Zambia, Zimbabwe and Kenya with a maximum total of 1266 children. In Zimbabwe, we will enroll 422 children.

WHAT WILL HAPPEN WHEN MY CHILD ENTERS THE STUDY?

A Research Nurse will approach you in hospital if your child has been admitted with SAM, and has been started on Plumpy'nut. We will check that you and your child can take part in the study, then ask for your written consent after you have had a chance to read this document and ask any questions. We will ask you some questions about your home situation, your child's health, and your own health. We will repeat

measurements of your child's weight, height, and upper arm size. We will collect a maximum of 6mL of blood from your child, which is just over one teaspoon of blood, and a stool sample. These samples will be sent to a laboratory to measure how well your child is recovering from malnutrition at the time s/he is leaving hospital.

We will start the interventions while your child is in hospital and continue them for 12 weeks. We will ask you to bring your child to our study clinic after 2 weeks, 4 weeks, 6 weeks, 8 weeks, 12 weeks, and 24 weeks, so that we can give treatments, including all the standard care treatments your child needs. At these visits we will measure your child's weight, height, and upper arm size, to see how the treatment is working, and collect medical information about your child by asking you some questions and reviewing his/her medical records. If you are placed in an arm with the psychosocial intervention then we will also see you at 1 week, 3 weeks and 5 weeks after leaving hospital to deliver the play and problem-solving therapy. Before leaving hospital, on week 2, and week 12 we will collect a maximum of 6mL of blood from your child, which is just over one teaspoon of blood, and a stool sample from your child. Each of these visits will last about an hour. The length of time that your child will be in the study is 24 weeks in total, i.e., 6 months. You may choose a 'buddy', perhaps a relative or friend, to get involved and support you during the study. Because it is really important that you attend all study visits, we may contact you by phone to remind you of visits or to collect information if you missed a study visit. If we cannot reach you by phone, we may come to visit you at your home, with your permission.

PROCEDURES AND DURATION

All children in the study will get, at a minimum, the standard care of treatment offered to children leaving hospital after treatment of SAM. There are new treatment packages that we have carefully designed and would like to provide in addition to standard care, so that we can see if adding them improves recovery of children. We do not know if these new treatments are better than the existing treatments, so we need to compare them in a randomised clinical trial. This means that the choice of treatments is made randomly by a computer, by chance (like the flip of a coin). Out of every 6 children, for 2 of them the treatment will remain the same as the standard care we give now, and the other 4 will have the current treatment PLUS one or all of the new treatments. It is very important that you understand these two principles of a randomized trial:

1. We genuinely do not know which treatment is best, or if they are better or worse than what we do already.
2. Children will be allocated a treatment by chance.

TREATMENTS

Your child will be placed into one of the following five (5) groups. Over the course of the study, twice as many children will be placed in the 'Standard of Care' group as compared to the other groups. All treatments will be given for 12 weeks:

1. **Standard of Care.** This will be the usual treatment that we know is the best we can currently provide to children with SAM. This includes ready-to-use therapeutic food (Plumpy'nut), and, if your child is living with HIV, antiretrovirals (ARVs) and cotrimoxazole to prevent infections; your child's doctor may also have started other medicines in hospital, depending on any other problems s/he identified in your child. We will monitor your child regularly in clinic and you can also contact us if you have concerns.
2. **Antibiotics.** We will find out whether a package of antibiotics given for 12 weeks prevents infections and helps children recover at home. This will involve taking several liquids, or tablets which can be dissolved in water, to prevent infections like pneumonia and TB. These medications are well tolerated in most children, although all medicines can have side-effects. The dose will be based on the weight of your child. We will monitor your child regularly in clinic and you can also contact us if you have concerns.
3. **Reformulated Ready-to-use-therapeutic food (RUTF).** Children in this group will receive Plumpy'nut with a slightly different recipe, which is designed to be more easily absorbed and to help the body's digestion of nutrients. The number of sachets will be the same as regular Plumpy'nut, and it will look very similar, although it may have a slight difference in flavour. Plumpy'nut is already given to

children leaving hospital and is well-tolerated. We will monitor your child regularly in clinic and you can also contact us if you have concerns.

4. **Psychosocial.** We have studied how the home environment and the mental health of caregivers can affect how children recover following SAM. We have developed a package including play therapy, and the Friendship Bench, which provides one-to-one problem-solving therapy, and peer support groups. You will meet a facilitator every week for 6 weeks to talk about any challenges you may be facing, and to help your child recover through play, and learn more about ways to care for your child when they are sick. After the 3rd week, you will be invited to join a support group with other caregivers in this trial, to talk to each other about your shared challenges, and to start activities together. It is possible that some discussion questions or topics will make you feel uncomfortable or unhappy. The interventions that we will be using are designed to help you work through those feelings and to find solutions. However, if you don’t want to discuss something, you do not have to. We will monitor your child regularly in clinic and you can also contact us if you have concerns.
5. **Combined.** Children in this group will receive ALL of these interventions (antibiotics, reformulated RUTF and psychosocial), to see whether giving all interventions together is feasible and provides the best recovery for children after SAM. We will monitor your child regularly in clinic and you can also contact us if you have concerns.

ARE THERE ANY RISKS TO PARTICIPATING IN THIS STUDY?

This is a research project, and these treatments are extra to what is usually given to children with SAM. All treatments can have some risks, as described above, but we have designed them to minimize these risks. However, the possibility remains that some children might experience adverse reactions to treatments. We can promise you that we will watch out carefully for such reactions and that we will provide the best possible care in the event that a reaction does occur. Blood taking will happen three times during the study and can be uncomfortable for children, but we will do our best to minimize this. There is a possibility of bruising and swelling when giving blood, and a very small risk of infection. We do not expect collection of stool samples to cause discomfort for your child. Some of the questions we ask could make you feel uncomfortable, and you should feel free to skip any questions that you do not wish to answer.

WHAT ARE THE BENEFITS TO TAKING PART IN THIS STUDY?

Four of these treatment options are new and we do not know at this stage whether they will work and we cannot therefore guarantee any benefits for your child. Your child will continue to receive the best possible care for SAM and frequent monitoring by our team in our dedicated study clinic. You will be able to contact the team if you have questions or concerns in between the regular visits. We hope that the new treatments will speed up children’s recovery after hospitalization.

We hope that our learning from this trial will benefit children with SAM in the future. At the end of the trial we will share our results with you.

WHAT ARE THE COSTS TO ME/MY CHILD?

You will not receive payment for participating in this trial. We will reimburse costs of USD10 when we ask you to bring your child to the study clinic. We will provide light refreshments at each study visit.

ALTERNATIVE PROCEDURES OR TREATMENTS

Your child will continue to receive the current standard of care for children with severe acute malnutrition whether or not s/he participates in the study.

WHAT ABOUT PROTECTING MY CHILD’S PERSONAL INFORMATION?

During the research we collect information about you and your child. We store the information on computers and remove your name and your child’s name and any other ways of being identified, so that there is no way of telling who the information refers to. Instead, you will be given a unique study ID. All our research records are stored securely in locked cabinets and password-protected computers and are accessed only by authorized persons.

The personally identifiable information you provide will be used to contact you for scheduling of visits, for safety assessments and verification of consent through signed consent forms if applicable.

All information you give us is kept confidential. This means that we are not allowed to tell anyone about you or your child, even if they are your friends or family. Your child's medical notes and study information will be available to study staff and may also be seen by other independent people authorized to ensure that the study is being properly carried out such as the Medical Research Council of Zimbabwe and the Medicines Control Authority of Zimbabwe. We will share anonymized individual and summary information we collect or generate with the sponsor, Queen Mary University of London, in ways that do not reveal your identity or that of your child. The study site will keep personally identifiable information about you from this study for 25 years after the study has finished in accordance with applicable Data Protection Requirements. You have the right to access the personal data we hold that pertains to you and your child, to object to or make corrections to the processing of all or part of the personal data.

Queen Mary University of London is responsible for ensuring that staff involved in the trial adhere to the safe and proper use of any personal information you provide. You can contact the research team to facilitate any contacts with the Queen Mary University of London's Data Protection Officer for any further information about how your data will be managed.

WHAT IF I DON'T WANT MY CHILD TO TAKE PART IN THE STUDY OR I WANT TO WITHDRAW LATER?

Participation in this study is voluntary. It is completely up to you whether or not your child participates, or if you join the trial but decide to withdraw later. If you decide for your child not to participate, it will not affect the treatment your child receives now or in the future. Whatever decision you make, it will not affect your relationship with the staff caring for your child. If you do not come back for any study visits after enrolling, we will exit you from the trial.

OFFER TO ANSWER QUESTIONS

Before you sign this form, please ask any questions on any aspect of this study that is unclear to you. You may take as much time as necessary to think it over.

IN THE EVENT OF INJURY

In the unlikely event that your child is harmed as a result of this trial, Queen Mary University of London has agreed that you will be compensated, provided that, on the balance of probabilities, an injury was caused as a direct result of the intervention or procedures your child received during the course of the study. These special compensation arrangements apply where an injury is caused to your child that would not have occurred if s/he were not in the study. These arrangements do not affect your right to pursue a claim through legal action.

WHAT HAPPENS TO SAMPLES COLLECTED?

We will use the samples to test how well your child is recovering from malnutrition, by measuring nutrients, the health of the gut and the healthy bacteria living in the gut, and how well the immune system is working.

Most of the research tests that will be done on the sample will be done in this country. However, for some tests that cannot be done in the country, samples will be sent to laboratories overseas.

The samples will be destroyed in line with regulations around sample storage, usually after 25 years.

CONTACTS AND QUESTIONS

If you have any questions or concerns about the study or how it is conducted, you can contact Dr Mutsa Bwakura Dangarembizi, the Principal Investigator, on +263 (242) 306028/306654. If you have any questions concerning this study or consent form beyond those answered by the investigator, including questions about the research, your rights as a research participant or research-related injuries; or if you feel that you have been treated unfairly and would like to talk to someone other than a member of the research team, please feel free to contact the Medical Research Council of Zimbabwe on telephone +263 (242) 791193/791792/08644073772 or cellphone +263 784 956 128.

You may also want to visit the Medical Research Council of Zimbabwe offices located at
20 Cambridge Road
Avondale
Harare

YOU WILL BE GIVEN A COPY OF THIS CONSENT FORM TO KEEP.

THIS FORM IS NOT VALID WITHOUT THE IRB STAMP OF APPROVAL

Signed copies of this consent form must be 1) given to the study participant and 2) placed in the study participant's research record

No. 16 Lauchlan Avenue
Meyrick Park, Mabelreign
Harare, Zimbabwe
Tel:+263 (242) 306028 / 306654
E-mail: administrator@zvitambo.com

An adaptive multi-arm trial to improve clinical outcomes among children recovering from complicated severe acute malnutrition (Co-SAM)

Principal Investigator: Prof Mutsa Bwakura Dangarembizi

Phone number(s) +263 (242) 306028/306654

AUTHORIZATION

You are making a decision about whether or not to allow your child to participate in this study. Your signature indicates that you have read and understood the information provided above, have had all your questions answered, and have decided for your child to participate.

You are confirming you understand that allowing your child to take part in the research will likely include shipment of your child's samples abroad. You understand that you can change my mind at any stage and it will not affect you or your child in any way.

Child's Name: (please print) ____________________
(first name, last name)

____
Full Name of Caregiver *(first name, last name)*

Date: ☐☐ / ☐☐☐ / 2 0☐☐ Time: ☐☐:☐☐
day *month* *year*

Signature of Caregiver
or thumb print if unable to sign ____________________

Relationship of caregiver to the child: ____________________

Independent Witness: If the caregiver cannot sign for himself or herself, a witness to the consent process should sign on his or her behalf.

____________________ ____________________
Full Name of Witness (*first name, last name*) Signature of Witness

Relationship of witness to caregiver: ____________________

Date: ☐☐ / ☐☐☐ / 2 0☐☐ Time: ☐☐:☐☐
day *month* *year*

Person obtaining consent:

I confirm that I have personally explained the nature and extent of the planned research, study procedures, potential risks and benefits, and confidentiality of personal information.

____________________ ____________________
Name of person obtaining consent Signature of person obtaining consent
(first name, last name)

Date: ☐☐ / ☐☐☐ / 2 0☐☐
day *month* *year*
