# Supplementary material for: An adaptive multiarm randomised trial of biomedical and psychosocial interventions to improve convalescence following severe acute malnutrition in sub-Saharan Africa: Co-SAM trial protocol
Source: BMJ Open. 2025 May 24;15(5):e093758. doi: 10.1136/bmjopen-2024-093758 (PMC12104919; doi:10.1136/bmjopen-2024-093758)
Supplement: online supplemental file 2 [file bmjopen-15-5-s002.docx]

**Supplementary Table 1**

Standard RUTF formula versus reformulated RUTF formula

|  | **Standard RUTF (Plumpy’nut)** | | **Reformulated RUTF** | |
| --- | --- | --- | --- | --- |
| **Ingredient** | **Proportion** | **Grams per sachet*** | **Proportion** | **Grams per sachet*** |
| Dairy ingredients (skimmed milk powder and whey powder) | 25-35% | 23 - 32.2 | 20 – 30% | 18.4 – 27.6 |
| Peanuts | 20 - 30% | 18.4 – 27.6 | 10 – 20% | 9.2 – 18.4 |
| Palm oil | 5 – 15% | 4.6 – 13.8 |  |  |
| Sugar | 15 - 25% | 13.8 - 23 | 15 – 25% | 18.5 - 26 |
| Vitamins and minerals | 1- 3% | 0.9 – 2.8 | 1- 3% | 0.9 – 2.8 |
| Canola oil | 15-15% | 4.6 – 13.8 | 5 – 15% | 0.9 – 2.8 |
| Emulsifier | 1- 3% | 0.9 – 2.8 | 1- 3% | 0.9 – 2.8 |
| Coconut Oil |  |  | 10 – 20% | 9.2 – 18.4 |
| Whey protein hydrolysate |  |  | 5 – 15% | 4.6 – 13.8 |
| **Macro composition** | | | | |
| Protein (g/100g) | 14.4 |  | 15.5 |  |
| Hydrolysed protein as % of total protein | 0 |  | 38 |  |
| PDCAAS (Catch up growth, FAO  2017) (%) | 91 |  | 100 |  |
| Fat (g/100g) | 34.8 |  | 34.2 |  |
| Medium-chain triglycerides as %  of fat | 0 |  | 29.2 |  |
| n3 fatty acids (g/100g) | 0.76 |  | 0.61 |  |
| n6 fatty acids (g/100g) | 3.5 |  | 2.1 |  |
| Ratio n6/n3 | 4.6 |  | 3.4 |  |
| Carbohydrate (g/100g) | 43 |  | 42 |  |
| Total energy | 543 Kcal/100g |  | 543 Kcal/100g |  |
