# Supplementary material for: An adaptive multiarm randomised trial of biomedical and psychosocial interventions to improve convalescence following severe acute malnutrition in sub-Saharan Africa: Co-SAM trial protocol
Source: BMJ Open. 2025 May 24;15(5):e093758. doi: 10.1136/bmjopen-2024-093758 (PMC12104919; doi:10.1136/bmjopen-2024-093758)
Supplement: online supplemental file 3 [file bmjopen-15-5-s003.docx]

**Supplementary Table 2**

| In hospital | | | | Study clinic (time-points are weeks post-randomisation) | | | | | | | | | | |
| --- | --- | --- | --- | --- | --- | --- | --- | --- | --- | --- | --- | --- | --- | --- |
| **Procedures** | **Screening** | **Consent** | **Baseline/randomisation** | **(Week 1)** | **Week 2** | **(Week 3)^a^** | **Week 4** | **(Week 5)^a^** | **Week 6** | **Week 8** | **Week 12** | **Week 24** | **Unscheduled visit** |  |
| Routine medical care | X | X | X | X | X | X | X | X | X | X | X | X | X |  |
| Screening^b^ | X |  |  |  |  |  |  |  |  |  |  |  |  |  |
| Written informed consent |  | X |  |  |  |  |  |  |  |  |  |  |  |  |
| Locator/contact information |  |  | X | X | X | X | X | X | X | X | X | X |  |  |
| Baseline questionnaire |  |  | X |  |  |  |  |  |  |  |  |  |  |  |
| TB diagnostics^c^ |  |  | X |  |  |  |  |  |  |  |  |  |  |  |
| HIV testing^d^ |  |  | X |  |  |  |  |  |  |  |  |  |  |  |
| ART and cotrimoxazole supply (if HIV-positive)^e^ |  |  | X |  | X |  | X |  | X | X | X | X |  |  |
| RUTF supply^f^ |  |  | X |  | X |  | X |  | X | X | X |  |  |  |
| IMP supply (antimicrobial arms) |  |  | X |  | X |  | X |  | X | X |  |  |  |  |
| Anthropometry^g^ |  |  | X |  | X |  | X |  | X | X | X | X |  |  |
| Stool collection^h^ |  |  | X |  | X |  |  |  |  |  | X |  |  |  |
| Lithium heparin blood collection (max 4mL)^i^ |  |  | X |  | X |  |  |  |  |  | X |  |  |  |
| Safety blood assessment (max 2mL)^j^ |  |  | X |  | X |  |  |  |  |  |  |  | X |  |
| Follow-up questionnaire |  |  |  |  | X |  | X |  | X | X | X | X |  |  |
| Study interventions (arm-specific)^k^ |  |  | X | X | X | X | X | X | X | X | X |  |  |  |
| Study intervention adherence check |  |  |  | X | X | X | X | X | X | X | X |  |  |  |

^a^Weeks 1, 3 and 5 are intervention visit for caregiver-child pairs in the psychosocial arms only. Visit windows for each visit are: Week 1 (week 0-2), 2 (week 1-3), 3 (week 2-4), 4 (week 3-5), 5 (week 4-6), 6 (week 5-7), 8 (week 7-11), 12 (week 11-week 22), and 24 (week 22-48).

^f^All children receive RUTF for a minimum of 2 weeks (standard RUTF in all arms except the reformulated RUTF arm); thereafter, continuation in all arms is based on child anthropometry, until WHZ>-2 and MUAC >12.5cm, and children are oedema-free since the last visit, for a maximum of 16 weeks of RUTF. The same criteria will be used for the reformulated RUTF arm.

^g^Weight, length/height, MUAC, oedema assessment. At baseline, head circumference will also be measured.

^h^ Allowable window for stool collection: baseline (up to 72h post randomisation); 2 weeks (+/- 1 week); and 12 weeks (week 11-week 22).

^j^Serum alanine transaminase (ALT) will be measured in all arms at baseline and at 2 weeks. If the ALT is abnormal, a complete liver function test panel will be requested.

^k^Interventions are given for 12 weeks in total, and will start as soon as possible after randomisation. For children who have to interrupt interventions (e.g. due to drug toxicity, or admission to hospital) the intervention will be restarted as soon as possible after interruption to provide a 12-week intervention in total.
