# Supplementary material for: An adaptive multiarm randomised trial of biomedical and psychosocial interventions to improve convalescence following severe acute malnutrition in sub-Saharan Africa: Co-SAM trial protocol
Source: BMJ Open. 2025 May 24;15(5):e093758. doi: 10.1136/bmjopen-2024-093758 (PMC12104919; doi:10.1136/bmjopen-2024-093758)
Supplement: Supplementary file 4 [file bmjopen-2024-093758.reviewer_comments.pdf]

PEER REVIEW HISTORY

BMJ Open publishes all reviews undertaken for accepted manuscripts. Reviewers are asked to complete a checklist review form and are provided with free text boxes to elaborate on their assessment. These free text comments are reproduced below.

ARTICLE DETAILS

Title (Provisional)

An adaptive multi-arm randomised trial of biomedical and psychosocial interventions to improve convalescence following severe acute malnutrition in sub-Saharan Africa: Co-SAM trial protocol

Authors

Bwakura-Dangarembizi, Mutsa; Amadi, Beatrice; Singa, Benson O; Muyemayema, Sofia; Ngosa, Deophine; Mwalekwa, Laura; Ngao, Narshion; Kazhila, Lydia; Mutasa, Batsirai; Mpofu, Eddington; Mudawarima, Louisa; Gonzales, Gerard Bryan; Mudzingwa, Shepherd; Mutenda, Mukumbi; Keter, Lucia K; Mutasa, Kuda; Njunge, James M.; Jones, Helen; Phiri, Tracy Naomi; Mudibo, Evans; Chulu, Nivea; Majo, Florence D; Chasekwa, Bernard; Tembo, Aaron; Nyabinda, Churchil; Oduol, Chris; Sauramba, Virginia; Tavengwa, Naume V; Langhaug, Lisa; Cordani, Isabella; Smuk, Melanie; Jaki, T; Ntozini, Robert; Walson, Judd; Tickell, Kirkby D; Berkley, James; Kelly, Paul; Prendergast, Andrew

---

VERSION 1 - REVIEW

**Reviewer**	**1**
**Name**	**Daures, Maguy**
**Affiliation (INSERM)**	**National Institute for Health and Medical Research**
**Date**	**05-Dec-2024**
**COI**	**None**

Review of the BMJ Open Article: "An adaptive multi-arm randomised trial of combined biomedical and psychosocial interventions to improve convalescence following severe acute malnutrition: Co-SAM trial protocol"

General Assessment

The article presents a protocol for an important and relevant study addressing the recovery of children following complicated severe acute malnutrition (SAM). The design is innovative, utilizing an adaptive multi-arm randomized trial (MART) to test biomedical and psychosocial interventions. The protocol is well-written, with clear objectives and a comprehensive methodological approach. However, several aspects require clarification and refinement for full compliance with the SPIRIT guidelines and to strengthen its scientific rigor.

Of note : The page numbers indicated in the Spirit checklist are mostly shifted by 1.

Assessment using the SPIRIT Checklist

Item 3 : The version is not mentioned in the manuscript and its history or any amendments are not explicitly discussed. Adding this would improve transparency.

Item 4: Funding sources are disclosed (page 22 and not 23), but the potential influence of funders on study design and conduct is not fully elaborated.

Item 6a: The background provides sufficient detail about SAM and its challenges. However, there is no mention of antibioresistance, which is critical when antibiotics are a component of the interventions. Including a discussion on the prevalence of antimicrobial resistance in the study regions would be interested.

Item 7 : The primary and secondary objectives are stated, but secondary objectives ii) could be more explicit to explain what mechanism will be studied.

Item 9: This study sites are not detailed in the protocol. Given the proposed intervention, it is important to provide contextual information about the countries and wards selected for the trial. Specifically, the authors should include data on the prevalence of acute malnutrition and infectious diseases such as TB to better justify the inclusion of the antimicrobial arm.

Item 11:

Page 11 line 44 : « The other two arms will be introduced when the reformulated RUTF has been developed » Authors need to provide details about its current status or timeline for availability.

Page12 line 47 : « trial arms will be introduced once each intervention is ready for testing ». Introducing trial arms as interventions become ready raises concerns about the seasonal variability of malnutrition. The protocol should ensure equal distribution of participants across the seasonal peaks as it is well known that mortality is higher during this season.

p 16 line 23 : « RUTF will be supplied in all arms for at least 2 weeks ». Acute malnutrition programs usually involve weekly visits. Autors should specify if the bi-weekly visits are proposed as part of the standard protocol or specifically for trial? If not, this should be explicitly specified, as it represents a modification of the standard protocol. Weekly outpatient follow-up for complicated SAM children discharged from hospitalization could be justified and should be considered by the authors.

Item 12: If I understand correctly, mortality and hospitalization are evaluated throughout 24 weeks, while nutritional recovery is assessed at Week 12. This distinction is made, but it could be more explicit in the primary outcome definition.

Relapses represent a significant challenge to the efficiency and mid-term impact of acute malnutrition programs. Measuring whether the intervention reduces relapses between Week 12 and Week 24, when relapses are typically most frequent, would provide valuable insights.

Item 13 : p21 lines 13-16, In the section « time frame and study status », the authors state, "The trial started enrolment in Zimbabwe and Zambia in July 2024, and is expected to start enrolment in Kenya in October 2024." This sentence needs clarification if the trial is already

started? If so, this should be clearly explained, including details on how many children have been enrolled to date.

Item 14: The calculation is clear but does not specify if it applies to individual countries or across all sites. Clarification is needed.

Item 17a: The randomization method is described, but there is inconsistency between the SPIRIT checklist (which inform that the trial is blinded) and the randomization paragraph (which does not mention blinding). If blinding is not used, this needs to be corrected.

Item 20a: The statistical methods are clearly described, but the authors do not mention any per-protocol analysis, which would be justified in a trial of this nature. Clarification is needed.

Items 25, 29, 30, and 31c should be clearly specified in the protocol article according to the SPIRIT checklist.

**Reviewer**	**2**
**Name**	**PHELAN, Kevin**
**Affiliation**	**ALIMA**
**Date**	**08-Jan-2025**
**COI**	**From 2019-2022, Kevin Phelan served on the Social Purposes Advisory Commission of Nutriset, a main producer of lipid-based nutrient supplement products.**

This is a very interesting and important study with a strong design. I have already recommended accepting because of the strength and clarity of the paper, but I did have a few questions and minor comments that I would like to share with the authors.

1. Page 7/41, line 39: coincident is misspelled

2. Page 10/41: Authors state "However, about 45-60% of calories in RUTF are supplied in the form of long-chain triglycerides, which may contribute to the metabolic stress, and hence to the poor outcomes among children with SAM." You may consider specifying complicated SAM here as current RUTF has very good outcomes for uncomplicated SAM.

3. "failed nutrition recovery" is one of the outcomes. I am assuming this would include the children categorized as 'non-responders' during 12 weeks of treatment. New research is showing that this is a poor category, as it is often used to mean 'treatment failure' even though there is likely a decent response to treatment by these kids but they simply fail to reach the threshold for recovery. Have you thought about some way to separate out the true treatment failures and make that part of the composite definition for the primary objective?

REF: Daures M, Hien J, Cazes C, Alitanou R, Saillet L, Séri B, Aly AAM, Maidadji O, Sanoussi A, Mahamadou A, Altmann M, Phelan K, Becquet R, Shepherd S. Factors associated with non-response and nutrional status of non-responders at 6-month post-discharge: a cohort study nested in a MUAC-based nutrition programme for acutely malnourished children in Mirriah, Niger. Front Public Health. 2024 Aug 14;12:1357891. doi: 10.3389/fpubh.2024.1357891. PMID: 39206006; PMCID: PMC11349736.

4. The definition of nutritional recovery used is WHZ>-2 AND MUAC >12.5 cm AND absence of oedema since the last study visit. I am not sure of the protocols in the countries here, but some countries' protocols state that nutritional recovery is achieved either by WHZ OR by MUAC criteria, and often times it is >=. And will this be one week only acheiving those thresholds or 2 consecutive visits?

5. In the manuscript you often times use the brand name Plumpy'nut. I would advise using the generic RUTF to refer to the current formulation and then something else for the reformulated RUTF (even "reformulated RUTF" is not too cumbersome).

6. Are there any plans to document the intervention costs? I think that would be very useful, but I would understand if not : a study cannot do everything all at once!!

In any event, I want to commend the entire team for designing such a strong study that could answer some important questions to improve kids' chances of surviving an episode of severe malnutrition that put them in hospital. Good luck to the whole team...

**Reviewer**	**3**
**Name**	**Kangas, Suvi T.**
**Affiliation**	**International Rescue Committee, Airbel Impact Lab**
**Date**	**13-Jan-2025**
**COI**	**None**

Congratulations to the authors for a well written study protocol describing a really interesting and important study testing different interventions to improve short term survival and health of children with SAM admitted to inpatient treatment and discharged to outpatient care to finish treatment. The use of an adaptive design is a welcome innovation and I appreciate how the authors have justified its use and the rules for adaptations following the interim analysis. The manuscript is generally clear and comprehensive. Thus my comments are minor.

General:

- Generally it would be good to use continuous line numbering, not one that starts again at each page.

Abstract:

- Please make sure it is clear that all arms receive the standard of care in addition to the interventions.

- Is intervention really provided for 12weeks regardless of recovery? Or only until recovery? I would assume RUTF is stopped when recovery is attained. Please clarify.

- Can you define recovery somewhere in the abstract.

Methods:

- Please specify that this is an individually randomized trial somewhere in the methods

- You are referring to figure 2 when talking about study contexts (page 10). But I don't think this actually speaks to that.

- Can you speak to how cross contamination is limited? Particularly for the psycosocial intervention which seems difficult to potentially contain seen you will have participants randomized to different arms in the same communities..

- What is the composition of the reformulated RUTF? It might be helpful to have a table that compares its nutritional composition against standard RUTF. Also, I'm curious to whether you have planned an acceptability trial before testing it or if you have planned to collect indicators on intake.

- Beyond transport reimbursement (or will it rather be a fixed amount you give to all?) is there any other compensation for the participants?

- Table 2 seems to only capture anthropometry & clinical outcomes but nothing on what will be done with stool and blood…

- Figure 1: I find this graph pretty confusing and am having hard time following what is the cause and the consequence here. The fact that you have put together mortality+readmission with impaired neurodevelopment seems off. I think you could create a better visualization to describe the framework. I think it would also be useful to potentially integrate the intervention target objectives in. Meaning, which intervention aims to tackle which problem(s).

- Figure 2: The copy-editing seems to gone wrong with the arrows. Additionally, you seem to be mixing 2 figures here: 1 on patient flow and the other on study flow. I would suggest you separate them out. The study flow might be interesting to depict on a timescale where you can also add the introduction of your 2 final arms.

- A description of the study settings is missing. What is the prevalence of SAM around these areas? What are the other epidemiological characteristics of the populations? Also, I'm wondering if you could specify how the study clinics are situated from the hospitals. I'm just worried that you will only be able to capture those living really close to the hospital (while those referred from further will not be able to attend outpatient clinics far from their home village)

- I'm surprised by the strictness of your recovery criteria: if I'm understanding correctly, you are saying that the child should reach WHZ>-2 and MUAC>125mm and absence of edema latest at week 8 (second last study visit) and again at week 12 (last study visit). I believe this is really short and you will be overestimating the number of failed nutritional recovery. Many programs report mean length of stays >8weeks for SAM in outpatient. For cases who have started as inpatients, this is probably even longer… Also, I'm wondering why you chose fortnightly visits instead of weekly ones (and stretching to 4week separation between 8 and 12w). The WHO guidelines suggest a weekly monitoring schedule for children with SAM and there is evidence to show that spacing out visit frequency negatively influences treatment outcomes, potentially due to worse adherence.. Also, WHO criteria for recovery is rather WHZ= or >-2 and MUAC = or >125mm etc…So wondering why you decided to use strictly >.

- I regret that you have not planned (or at least did not mention?) a formal cost-efficacy analysis of the interventions. One of the key problems with SAM treatment globally is that there is limited resources to actually support the treatment activities, including the purchase of the necessary RUTF. The cost of the product has also been increasing due to product innovations following clinical trials showing some beneficial outcomes with improved but more costly formulations. What this then means from the public health perspective is that less product is available for children meaning less children are treated. My worry is that all these novel formulations may indeed be clinically beneficial but will lead to ever increasing cost of product in the market undermining the public benefit. And I'm not even talking about more and more difficult production for local producers already struggling with the current formulations (if you would like to consider an equity lense).

VERSION 1 - AUTHOR RESPONSE

Reviewer: 1

General Assessment
The article presents a protocol for an important and relevant study addressing the recovery of children following complicated severe acute malnutrition (SAM). The design is innovative, utilizing an adaptive multi-arm randomized trial (MART) to test biomedical and psychosocial interventions. The protocol is well-written, with clear objectives and a comprehensive methodological approach. However, several aspects require clarification and refinement for full compliance with the SPIRIT guidelines and to strengthen its scientific rigor.

Of note : The page numbers indicated in the Spirit checklist are mostly shifted by 1.

Apologies for this. We have updated the SPIRIT checklist with the resubmission against the clean version of the updated manuscript to confirm that page numbers are correct.

Assessment using the SPIRIT Checklist

Item 3 : The version is not mentioned in the manuscript and its history or any amendments are not explicitly discussed. Adding this would improve transparency.

We have added this information to the revised SPIRIT checklist.

Item 4: Funding sources are disclosed (page 22 and not 23), but the potential influence of funders on study design and conduct is not fully elaborated.

We have updated the funding section, and included a statement on the role of Nutriset in the trial. This now reads: "This work was supported by the National Institute for Health and Care Research, UK (NIHR201813). AJP received funding from Wellcome (108065/Z/15/Z) and TJ also received funding from the UK Medical Research Council (MC_UU_00002/14). The funders had no role in trial design, implementation, manuscript preparation, or decision to publish. Queen Mary University of London (QMUL) and Nutriset S.A.S France collaborated to develop the reformulated Ready-to-Use Therapeutic Food (RUTF) specifically for this study. Under this collaborative agreement, Nutriset formulated and developed the new RUTF at its own expense while the production and supply of both the standard Plumpy'nut® and the reformulated RUTF were funded by the grant from NIHR to

QMUL. Nutriset will not participate in the conduct or reporting of the trial, and QMUL makes no claim in any manner to any rights in Nutriset products."

Item 6a: The background provides sufficient detail about SAM and its challenges. However, there is no mention of antibioresistance, which is critical when antibiotics are a component of the interventions. Including a discussion on the prevalence of antimicrobial resistance in the study regions would be interested.

Thank you, we have added the following comment with citations, to the background section: "Resistance to azithromycin among pathogens in east and Southern Africa remains relatively low, but broader use as a public health intervention increases antimicrobial resistance."

We also discuss the antimicrobial strategy and the risk of promoting antimicrobial resistance in the Risks and Benefits section: "There are several reasons why this trial will test universal prophylaxis, rather than targeted prophylaxis in a subgroup of children at high risk of infections. First, the population risk for mortality and readmission in children with SAM is extremely high. Second, we are currently unable to identify the children at highest risk of mortality following hospitalisation, meaning it is not possible to identify a subgroup most likely to benefit. Third, children are already treated in a targeted manner for symptomatic infections which can be diagnosed in the clinic, yet mortality remains unacceptably high, meaning new preventive approaches are required. Fourth, the trial is testing a pragmatic approach which could easily be scaled up. Finally, this approach has been safely tested in other trials with a very similar antimicrobial bundle[17]. If we find evidence of efficacy, the embedded mechanistic studies using biological samples may enable us to identify a subgroup of children to target in the future. At a population level, more widespread use of antibiotics must be balanced against the risk of promoting antimicrobial resistance (AMR). The recent MORDOR trial of azithromycin, which was associated with modest increases in AMR to macrolides[32], led to a WHO recommendation that mass administration of azithromycin was justifiable in settings of high child mortality[33]. Given the high-risk population of children with SAM (10% mortality over 12 months[5]), we believe this is a justifiable risk."

Item 7 : The primary and secondary objectives are stated, but secondary objectives ii) could be more explicit to explain what mechanism will be studied.

Thank you for this suggestion; we have amended to now read: "ii) to define the mechanism of action of the individual and combined intervention packages, through process evaluation to document fidelity of implementation and uptake of the psychosocial intervention, and laboratory substudies to explore the impact of the biomedical interventions on metabolic and inflammatory pathways."

Item 9: This study sites are not detailed in the protocol. Given the proposed intervention, it is important to provide contextual information about the countries and wards selected for the trial. Specifically, the authors should include data on the prevalence of acute malnutrition and infectious diseases such as TB to better justify the inclusion of the antimicrobial arm.

Thank you for this suggestion. We have now added a new section called 'Trial setting' which explain all nine sites in detail, to provide context to the trial. Unfortunately, there are no good national data on TB prevalence among children in these settings, but we added details about the prevalence of wasting. This section reads: "The Co-SAM trial is being undertaken at nine sites in three countries. In Zimbabwe, where the prevalence of wasting is 2%[29], three hospitals in Harare

(Sally Mugabe Children's Hospital, Parirenyatwa Hospital, and Chitungwiza Central Hospital) serve a predominantly urban and peri-urban population, although some children are referred for inpatient care from rural districts. In Zambia, the University Teaching Hospital Children's Hospital (UTHCH) in Lusaka provides secondary level services to the city of Lusaka (population 1.9 million, census 2022) and tertiary level services to the whole country (population 19 million). The child malnutrition ward in UTHCH provides inpatient care for children with complicated SAM, most of whom are drawn from high-density urban and peri-urban residential areas of the city. Matero Level 1 hospital provides primary and secondary level care for the high-density areas of north-west Lusaka. The prevalence of SAM in Zambia was 1.5% in 2018[30]. There are four hospitals in Kenya: Migori County Hospital and Homa Bay County Teaching and Referral Hospital serve a rural area in Western Kenya; Coast General Teaching and Referral Hospital, Mombasa serves an urban and peri-urban population on the coast; and Mbagathi sub-County Hospital predominantly serves a population living in informal settlements in Nairobi. The prevalence of acute malnutrition among under-5 children in Kenya in 2022 was 4.9%[31]."

Item 11:
Page 11 line 44 : « The other two arms will be introduced when the reformulated RUTF has been developed » Authors need to provide details about its current status or timeline for availability.
The new RUTF formulation has now been finalised and is being produced in February 2025, with product expected to be introduced in each country in May 2025. We have added details on the production process to the trial rationale section, which now reads: "The product underwent research and development by the manufacturer (Nutriset, France), in collaboration with the trial investigators, to produce the final composition shown in Supplementary Table 1… The reformulated RUTF is expected to go into production in February 2025, and be introduced into the trial in May 2025."

We have updated the randomization section to read: "The other two arms will be introduced when the reformulated RUTF has been developed (anticipated May 2025), and the ratio will be amended".

Page12 line 47 : « trial arms will be introduced once each intervention is ready for testing ». Introducing trial arms as interventions become ready raises concerns about the seasonal variability of malnutrition. The protocol should ensure equal distribution of participants across the seasonal peaks as it is well known that mortality is higher during this season.

The trial will enrol over 2-3 years in total, with a concurrent standard care arm across all three countries, meaning enrolment will cover several seasons for both intervention and control arms. Even for arms that are introduced later in the trial, enrolment will occur throughout the year over several seasons.

p 16 line 23 : « RUTF will be supplied in all arms for at least 2 weeks ». Acute malnutrition programs usually involve weekly visits. Autors should specify if the bi-weekly visits are proposed as part of the standard protocol or specifically for trial? If not, this should be explicitly specified, as it represents a modification of the standard protocol. Weekly outpatient follow-up for complicated SAM children discharged from hospitalization could be justified and should be considered by the authors.

Thank you for this important comment. Trial visits are mandated at weeks 2, 4, 6, 8, 12 and 24 to conduct trial-specific procedures and to collect data. However, standard care (common to all trial arms) allows the trial team to review children more frequently for clinical care, and this is determined on a case-by-case basis. Local practice across the nine sites outside of a trial setting

varies considerably, and although weekly follow-up is recommended in normative international guidelines, it is not always deployed in all sites. Our trial teams comprise experienced clinicians who will provide best available standard care based on local practice. In fact, our experience is that within a trial, care is often better than is possible in local Ministry of Health clinics, and our current follow-up rate (>95%) in Zimbabwe and Zambia is a testament to this. We have added an extra line to the follow-up section of the protocol which reads: "Children are seen for additional clinical visits as determined by the trial team."

Item 12: If I understand correctly, mortality and hospitalization are evaluated throughout 24 weeks, while nutritional recovery is assessed at Week 12. This distinction is made, but it could be more explicit in the primary outcome definition. Relapses represent a significant challenge to the efficiency and mid-term impact of acute malnutrition programs. Measuring whether the intervention reduces relapses between Week 12 and Week 24, when relapses are typically most frequent, would provide valuable insights.

All outcomes are assessed through to week 24, including nutritional recovery. If a child therefore relapses between week 12 and week 24, this will contribute to the primary outcome of the trial. This is stated in the footnote to Table 2, where failed nutritional recovery is "Defined as either: i) Persistent WHZ<-2 or MUAC<12.5cm or bilateral pedal oedema at week 12; or ii) WHZ<-2 or MUAC<12.5cm or bilateral pedal oedema at any time between baseline and week 24 post-randomisation in a child who had previously recovered". However, the wording of our follow-up section in the main manuscript was unclear and we have updated this to read: "If the child has not achieved nutritional recovery by week 12, or relapses before week 24 following nutritional recovery, s/he will be defined as reaching the primary outcome (failed nutritional recovery; see Table 2)…"

Item 13 : p21 lines 13-16, In the section « time frame and study status », the authors state, "The trial started enrolment in Zimbabwe and Zambia in July 2024, and is expected to start enrolment in Kenya in October 2024." This sentence needs clarification if the trial is already started? If so, this should be clearly explained, including details on how many children have been enrolled to date.

The reviewer is correct, that the trial started in Zimbabwe and Zambia in July 2024, and is expected to start in Kenya in March 2025. This section now reads: "The trial started enrolment in Zimbabwe and Zambia in July 2024, and is expected to start enrolment in Kenya in March 2025. By 20 February 2025, a total of 150 children had been enrolled to the trial. The estimated date of last participant enrolment is December 2027."

Item 14: The calculation is clear but does not specify if it applies to individual countries or across all sites. Clarification is needed.

We have updated this to read: "For the primary analysis, a total sample size of 1266 participants across all three countries, with a final ratio of 2:1:1:1:1 in favour of standard-of-care…"

Item 17a: The randomization method is described, but there is inconsistency between the SPIRIT checklist (which inform that the trial is blinded) and the randomization paragraph (which does not mention blinding). If blinding is not used, this needs to be corrected.

The trial is not blinded, as stated in the Methods section: "There will be no blinding or placebo as each trial arm has very different components. "

We cannot find the comment the reviewer mentions about blinding in the SPIRIT checklist, but we have clarified the updated item 17a to read: "The trial is not blinded, as stated on Page 11"

Item 20a: The statistical methods are clearly described, but the authors do not mention any per-protocol analysis, which would be justified in a trial of this nature. Clarification is needed.

Thank you for spotting this omission. Our protocol does indeed state that a per protocol analysis will be undertaken. We have updated this section of the manuscript to read: "The primary analysis will include all participants as randomised ("intention-to-treat"), irrespective of whether they received their allocated treatment. Both treatment effects and 95% confidence intervals will be reported for all primary, secondary and subgroup analyses. The per protocol analysis is a secondary analysis and will include all children without major protocol violation who had the primary outcome measured."

Items 25, 29, 30, and 31c should be clearly specified in the protocol article according to the SPIRIT checklist.

Thank you; we have now addressed each item as follows:

Item 25:
"Any protocol amendments will only be implemented after approval by regulators in each country and the Sponsor."

Item 29
"The final trial dataset will be made open access at ClinEpiDB after the analyses in the protocol have been reported."

Item 30
The standard care arm already described ancillary care, stating: "All children living with HIV will start ART and cotrimoxazole according to national guidelines. All medications required for standard care (e.g. antibiotics, TB treatment) will be provided. Children will be seen in a dedicated study clinic, with medical care provided as needed, and will not additionally need to attend their local outpatient therapeutic programme."

We have added text to the follow-up section: "No trial interventions will be provided beyond week 24. If children require ongoing care they will be linked to local Ministry of Health services."

Item 31c
We had already shown where the protocol is made available, in the Trial Design section: "The trial protocol and case report forms are available at https://osf.io/824av/."

We have added the following to the analysis section: "Statistical code for the main analyses will be made available with the primary publication. The final trial dataset will be made open access at ClinEpiDB after the analyses in the protocol have been reported."

Reviewer: 2
This is a very interesting and important study with a strong design. I have already recommended accepting because of the strength and clarity of the paper, but I did have a few questions and minor comments that I would like to share with the authors.

1. Page 7/41, line 39: coincident is misspelled

Thank you for spotting this; spelling has been corrected

2. Page 10/41: Authors state "However, about 45-60% of calories in RUTF are supplied in the form of long-chain triglycerides, which may contribute to the metabolic stress, and hence to the poor outcomes among children with SAM." You may consider specifying complicated SAM here as current RUTF has very good outcomes for uncomplicated SAM.

Thank you, this is an important point and we have added the word 'complicated'.

3. "failed nutrition recovery" is one of the outcomes. I am assuming this would include the children categorized as 'non-responders' during 12 weeks of treatment. New research is showing that this is a poor category, as it is often used to mean 'treatment failure' even though there is likely a decent response to treatment by these kids but they simply fail to reach the threshold for recovery. Have you thought about some way to separate out the true treatment failures and make that part of the composite definition for the primary objective?

REF: Daures M, Hien J, Cazes C, Alitanou R, Saillet L, Séri B, Aly AAM, Maidadji O, Sanoussi A, Mahamadou A, Altmann M, Phelan K, Becquet R, Shepherd S. Factors associated with non-response and nutronal status of non-responders at 6-month post-discharge: a cohort study nested in a MUAC-based nutrition programme for acutely malnourished children in Mirriah, Niger. Front Public Health. 2024 Aug 14;12:1357891. doi: 10.3389/fpubh.2024.1357891. PMID: 39206006; PMCID: PMC11349736.

The reviewer is correct that failed nutritional recovery includes children categorized as non-responders during 12 weeks of treatment, based on reaching anthropometric criteria for recovery defined in the trial protocol. However, our secondary and tertiary outcomes will assess change in MUAC and WHZ at weeks 4, 12 and 24, so this will allow us to address the important point the reviewer raises by exploring *change* in absolute values from baseline, instead of categorical outcomes of 'recovery' versus 'non-recovery'.

4. The definition of nutritional recovery used is WHZ>-2 AND MUAC >12.5 cm AND absence of oedema since the last study visit. I am not sure of the protocols in the countries here, but some countries' protocols state that nutritional recovery is achieved either by WHZ OR by MUAC criteria, and often times it is >=. And will this be one week only acheiving those thresholds or 2 consecutive visits?

The reviewer is correct that programmatic guidance often allows recovery to be defined by restoration of whichever anthropometric criterion was used to define SAM at programme entry. However, in this clinical trial we have defined a more strict outcome measure of nutritional recovery, which requires all three criteria to be met, because our goal is to ensure full convalescence through novel interventions, and not simply to meet criteria for programmatic discharge. Our prior work has highlighted the lack of nutritional recovery over a year following hospital discharge when these more strict criteria are used, and our trial aims to improve holistic recovery.

5. In the manuscript you often times use the brand name Plumpy'nut. I would advise using the

generic RUTF to refer to the current formulation and then something else for the reformulated RUTF (even "reformulated RUTF" is not too cumbersome).

Thank you; we have updated this to "standard RUTF", and where the interventional product is mentioned, we continue to use the term "reformulated RUTF".

6. Are there any plans to document the intervention costs? I think that would be very useful, but I would understand if not : a study cannot do everything all at once!!

We were intending to do health economics evaluation as part of the trial, but our health economist unfortunately left our team after the protocol was drafted. We therefore have a section in the trial protocol which outlines plans for 'within-trial' analysis and modelling to estimate incremental costs and outcomes over time, plus decision analytical modelling to estimate incremental cost-effectiveness, incorporating an estimate of disability-adjusted life years. However, we are currently seeking a new health economics collaborator and we are therefore uncertain currently whether we will be able to complete this work or not. Given this uncertainty, we prefer to leave it out of the current manuscript in case we are unable to do the work, but we have left it in the trial protocol.

In any event, I want to commend the entire team for designing such a strong study that could answer some important questions to improve kids' chances of surviving an episode of severe malnutrition that put them in hospital. Good luck to the whole team...

Many thanks for this comment!

<u>Reviewer: 3</u>
Congratulations to the authors for a well written study protocol describing a really interesting and important study testing different interventions to improve short term survival and health of children with SAM admitted to inpatient treatment and discharged to outpatient care to finish treatment. The use of an adaptive design is a welcome innovation and I appreciate how the authors have justified its use and the rules for adaptations following the interim analysis. The manuscript is generally clear and comprehensive. Thus my comments are minor.

We thank you for this comment.

General:
- Generally it would be good to use continuous line numbering, not one that starts again at each page.

We have updated the manuscript to include continuous line numbering as suggested.

Abstract:
- Please make sure it is clear that all arms receive the standard of care in addition to the interventions.

Thank you; we have updated this to read: "Standard-of-care, which is provided in all trial arms, includes ready-to-use therapeutic food (RUTF) and other management recommended in WHO guidelines."

- Is intervention really provided for 12weeks regardless of recovery? Or only until recovery? I would assume RUTF is stopped when recovery is attained. Please clarify.

The reviewer is correct that RUTF is provided until nutritional recovery. We have therefore updated the abstract throughout and this sentence now reads: "Standard-of-care, which is provided in all trial arms, includes ready-to-use therapeutic food (RUTF) until nutritional recovery (defined as weight-for-height Z-score >-2, MUAC >12.5cm and oedema-free for 2 weeks), and other management recommended in WHO guidelines." We have clarified that the antimicrobial intervention and psychosocial intervention are for 12 weeks.

- Can you define recovery somewhere in the abstract.

This has now been added as suggested (see response above).

Methods:
- Please specify that this is an individually randomized trial somewhere in the methods

This has now been added to the abstract and methods.

- You are referring to figure 2 when talking about study contexts (page 10). But I don't think this actually speaks to that.

We have referred to figure 2 in describing Co-SAM as a 5-arm randomised trial (trial design section). We have moved the study context up to a separate section which precedes this, in response to comments from reviewer 1.

- Can you speak to how cross contamination is limited? Particularly for the psycosocial intervention which seems difficult to potentially contain seen you will have participants randomized to different arms in the same communities.

The reviewer is correct that participants in the same community could be randomised to different interventions, but the catchment area of these referral hospitals is large and we do not anticipate much contamination between households living across a wide geographic area. If a caregiver has another child in the trial, the second child is randomised to the same arm as the first child to avoid contamination within families, as stated in the randomisation section: "If the caregiver has another child who has already been randomised in the trial, subsequent children will be automatically allocated to the same intervention."

- What is the composition of the reformulated RUTF? It might be helpful to have a table that compares its nutritional composition against standard RUTF. Also, I'm curious to whether you have planned an acceptability trial before testing it or if you have planned to collect indicators on intake.

The composition of the reformulated product is shown in Supplementary Table 1, compared to standard RUTF. We conducted a pilot affective sensory test among a group of children with SAM in Zambia prior to finalising the product, and found that it was as palatable as standard RUTF. We have updated the section on trial rationale to read: "The product underwent research and development by the manufacturer (Nutriset, France), in collaboration with the trial investigators, to produce the final composition shown in Supplementary Table 1. We conducted a pilot affective sensory test to explore the acceptability of the reformulated RUTF. A sachet of the reformulated RUTF was given to 25 children with complicated SAM ready to be discharged from hospital in Lusaka, Zambia. All children were eager to consume the reformulated RUTF, most finishing the sachet in less than one hour. Since we did not observe any sign of product rejection, the study team concluded that the RUTF was acceptable. A formal acceptability trial was not performed. The

reformulated RUTF is expected to go into production in February 2025, and be introduced into the trial in May 2025."

- Beyond transport reimbursement (or will it rather be a fixed amount you give to all?) is there any other compensation for the participants?

The details of reimbursement (fixed amount versus variable amount based on actual transport costs) differs across the nine sites, based on local and national guidelines, but in all sites includes compensation for time spent in the study clinic as well as transport costs. We have therefore updated this to read: "…with reimbursement provided for transport and time spent in the study clinic."

- Table 2 seems to only capture anthropometry & clinical outcomes but nothing on what will be done with stool and blood…

Table 2 shows the schedule of study visits, and when biological samples are collected. The details of how these samples will be used is provided in the laboratory analysis section of the manuscript, which states: "Planned laboratory analyses on archived samples include targeted and untargeted lipidomics, metabolomics, proteomics and biomarker assays. These analyses aim to elucidate key biological pathways linked to poor post-discharge convalescence including metabolic, lipid and hormonal perturbations, inflammation and enteropathy, and the impact of the trial interventions on these mechanisms."

- Figure 1: I find this graph pretty confusing and am having hard time following what is the cause and the consequence here. The fact that you have put together mortality+readmission with impaired neurodevelopment seems off. I think you could create a better visualization to describe the framework. I think it would also be useful to potentially integrate the intervention target objectives in. Meaning, which intervention aims to tackle which problem(s).

Thank you for these suggestions. The causes and consequences of malnutrition are often hard to disentangle, and our figure aims to capture the well-described cycle of malnutrition. We have now included an arrow from severe infection to clinical outcomes (since infections are frequently a cause of mortality and readmission). We have previously shown that inflammation underlies poor clinical outcomes also, which is why this pathway is highlighted (and this is another pathway that might be addressed by an immunomodulatory antibiotic such as azithromycin). We have removed neurodevelopment from the outcomes, and instead focused on our primary outcome of mortality, readmission and failed nutritional recovery. We have included, as suggested, the interventions in red font to show where each is hypothesied to act to improve clinical outcomes.

Figure 1

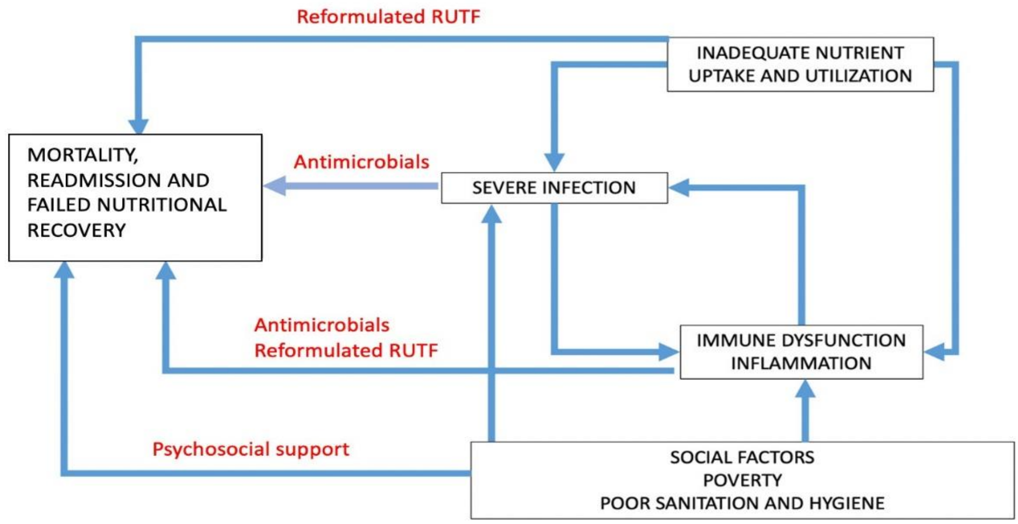

- Figure 2: The copy-editing seems to gone wrong with the arrows. Additionally, you seem to be mixing 2 figures here: 1 on patient flow and the other on study flow. I would suggest you separate them out. The study flow might be interesting to depict on a timescale where you can also add the introduction of your 2 final arms.

Thank you for spotting the copy-editing problem with the arrows which we have fixed. We appreciate the suggestion to split this into two figures but we believe this may be confusing to readers. We have deliberately tried to combine participant flow and study flow to explain how the adaptive nature of the trial works, and this figure is consistent with what is in our study protocol, so we would prefer to maintain the figure as it is.

- A description of the study settings is missing. What is the prevalence of SAM around these areas? What are the other epidemiological characteristics of the populations? Also, I'm wondering if you could specify how the study clinics are situated from the hospitals. I'm just worried that you will only be able to capture those living really close to the hospital (while those referred from further will not be able to attend outpatient clinics far from their home village)

Thank you. We have now included a new section on 'Trial setting' which provides this information. The catchment areas of the hospitals is quite large, but it is true that we will only enrol participants who are able to attend the study clinic for all their scheduled appointments. This is important to ensure we are able to deliver the intervention and capture outcome data in all children, but it is a limitation in terms of generalisability.

- I'm surprised by the strictness of your recovery criteria: if I'm understanding correctly, you are saying that the child should reach WHZ>-2 and MUAC>125mm and absence of edema latest at week 8 (second last study visit) and again at week 12 (last study visit). I believe this is really short and you will be overestimating the number of failed nutritional recovery. Many programs report mean length of stays >8weeks for SAM in outpatient. For cases who have started as inpatients, this is probably even longer… Also, WHO criteria for recovery is rather WHZ= or >-2 and MUAC = or >125mm etc…So wondering why you decided to use strictly >.

We have chosen more strict criteria than programmatic criteria for this trial, because our goal is to

ensure full convalescence through novel interventions, and not simply to meet criteria for programmatic discharge. Our prior work has highlighted the lack of nutritional recovery over a year following hospital discharge when these more strict criteria are used, and our trial aims to improve holistic recovery. However, our secondary and tertiary outcomes will assess anthropometric response to interventions at weeks 4, 12 and 24, including change in MUAC and WHZ (individually), so this will allow us to address absolute change in each anthropometric indicator, and to assess the trajectory of recovery, rather than only to use our strict primary outcome definition for recovery.

Also, I'm wondering why you chose fortnightly visits instead of weekly ones (and stretching to 4week separation between 8 and 12w). The WHO guidelines suggest a weekly monitoring schedule for children with SAM and there is evidence to show that spacing out visit frequency negatively influences treatment outcomes, potentially due to worse adherence..

Trial visits are mandated at weeks 2, 4, 6, 8, 12 and 24 to conduct trial-specific procedures and to collect data. However, standard care (common to all trial arms) allows the trial team to review children more frequently for clinical care, and this is determined on a case-by-case basis. Local practice across the nine sites outside of a trial setting varies considerably, and although weekly follow-up is recommended in normative international guidelines, it is not always deployed in all sites. Our trial teams comprise experienced clinicians who will provide best available standard care based on local practice. In fact, our experience is that within a trial, care is often better than is possible in local Ministry of Health clinics, and our current follow-up rate (>95%) in Zimbabwe and Zambia is a testament to this. We have added an extra line to the follow-up section of the protocol which reads: "Children are seen for additional clinical visits as determined by the trial team."

- I regret that you have not planned (or at least did not mention?) a formal cost-efficacy analysis of the interventions. One of the key problems with SAM treatment globally is that there is limited resources to actually support the treatment activities, including the purchase of the necessary RUTF. The cost of the product has also been increasing due to product innovations following clinical trials showing some beneficial outcomes with improved but more costly formulations. What this then means from the public health perspective is that less product is available for children meaning less children are treated. My worry is that all these novel formulations may indeed be clinically beneficial but will lead to ever increasing cost of product in the market undermining the public benefit. And I'm not even talking about more and more difficult production for local producers already struggling with the current formulations (if you would like to consider an equity lense).

These are all important considerations and we thank you for these comments. As mentioned in response to reviewer 2, we were intending to do health economics evaluation as part of the trial, but our health economist unfortunately left our team after the protocol was drafted. We therefore have a section in the trial protocol which outlines plans for 'within-trial' analysis and modelling to estimate incremental costs and outcomes over time, plus decision analytical modelling to estimate incremental cost-effectiveness, incorporating an estimate of disability-adjusted life years. However, we are currently seeking a new health economics collaborator and we are therefore uncertain currently whether we will be able to complete this work or not. Given this uncertainty, we prefer to leave it out of the current manuscript in case we are unable to do the work, but we have left it in the trial protocol. We agree that, if we find evidence of clinical efficacy, then implementation science is required to understand how these interventions would be scaled up.

We thank you once again for the comments on our paper and look forward to hearing from you.
